# Return rates for the use of ovarian tissue cryopreserved prior to gonadotoxic treatment as fertility preservation: a systematic review

**DOI:** 10.1093/hropen/hoaf068

**Published:** 2025-10-28

**Authors:** Anna Mathilde Yde, Lotte Berdiin Colmorn, Amalie Somuncu Johansen, Elisabeth Clare Larsen, Anja Pinborg, Kirsten Tryde Macklon

**Affiliations:** The Fertility Clinic, Department of Gynecology, Fertility and Obstetrics, Rigshospitalet—Copenhagen University Hospital, Denmark, Copenhagen; The Fertility Clinic, Department of Gynecology, Fertility and Obstetrics, Rigshospitalet—Copenhagen University Hospital, Denmark, Copenhagen; The Fertility Clinic, Department of Gynecology, Fertility and Obstetrics, Rigshospitalet—Copenhagen University Hospital, Denmark, Copenhagen; The Fertility Clinic, Department of Gynecology, Fertility and Obstetrics, Rigshospitalet—Copenhagen University Hospital, Denmark, Copenhagen; The Fertility Clinic, Department of Gynecology, Fertility and Obstetrics, Rigshospitalet—Copenhagen University Hospital, Denmark, Copenhagen; Department of Clinical Medicine, University of Copenhagen, Copenhagen, Denmark; The Fertility Clinic, Department of Gynecology, Fertility and Obstetrics, Rigshospitalet—Copenhagen University Hospital, Denmark, Copenhagen

**Keywords:** fertility preservation, ovarian tissue cryopreservation (OTC), autologous ovarian tissue transplantation (AOTT), return rates, gonadotoxic treatment, premature ovarian insufficiency (POI)

## Abstract

**STUDY QUESTION:**

What is the return rate for the use of ovarian tissue cryopreserved for fertility preservation prior to gonadotoxic treatment?

**SUMMARY ANSWER:**

The return rate for the use of ovarian tissue cryopreserved for fertility preservation prior to gonadotoxic treatment is modest, with most studies reporting rates of ≤5%.

**WHAT IS KNOWN ALREADY:**

A considerable number of years have passed since ovarian tissue cryopreservation (OTC) was first implemented for fertility preservation, and many studies now provide long-term follow-up data, including return rates. This allows for a more comprehensive evaluation of OTC as a fertility preservation option and of the characteristics of the population to whom it is offered.

**STUDY DESIGN, SIZE, DURATION:**

We searched PubMed, EMBASE, and the Cochrane Library for MeSH words, Emtree terms, and text words related to return rates for the use of ovarian tissue cryopreserved prior to gonadotoxic treatment up to March 2025, in the English language. There were no limitations regarding the year of publication.

**PARTICIPANTS/MATERIALS, SETTING, METHODS:**

Each study was screened independently by two reviewers who were blinded to each other’s choices. We included studies reporting the proportion of women who returned for autologous ovarian tissue transplantation (AOTT) among those who had undergone OTC. We excluded studies in languages other than English, abstracts without full text, letters to the editor, and case series involving fewer than 25 females undergoing OTC. Risk of bias was assessed using the Joanna Briggs Institute (JBI) Critical Appraisal Checklist for Cohort Studies. The overall certainty of evidence was assessed using GRADE (grading of recommendations, assessment, development, and evaluation).

**MAIN RESULTS AND THE ROLE OF CHANCE:**

Out of 2748 studies, 25 were included in the review. All studies were cohort studies that included from 26 to 2475 participants. Of the 25 studies, 18 reported a return rate of ≤5%, 6 reported a return rate of >5 to ≤10% while only 1 study reported a return rate of 14%. The overall quality of the studies in reporting the return rate for the use of cryopreserved ovarian tissue, as assessed using GRADE, was moderate.

**LIMITATIONS, REASONS FOR CAUTION:**

In most studies, follow-up occurred within the overall study period, and only one study reported a minimum follow-up duration. Since AOTT may occur several years after OTC, limited follow-up time may bias the results. Most studies included both pediatric patients and adults undergoing OTC, without accounting for participants who had not yet reached reproductive age or desired pregnancy when calculating return rates at the end of follow-up.

**WIDER IMPLICATIONS OF THE FINDINGS:**

The modest utilization rates found in this study emphasize the need for careful consideration to avoid subjecting women to potentially unnecessary and costly treatment. This highlights the need for future studies with longer follow-up time to assess patterns of tissue utilization in relation to diagnosis, treatment protocol, and age, thereby setting criteria in the selection of patients who would actually benefit from OTC.

**STUDY FUNDING/COMPETING INTEREST(S):**

The study is funded by the independent research fund Denmark (Grant ID 10.46540/4308-00130B). A.P. has received grants (payment to institution) and consultancy fees from Gedeon Richter, Ferring Pharmaceuticals, Merck A/S, Cryos; honoraria from Gedeon Richter, Ferring Pharmaceuticals, Merck A/S, and Organon; and support for attending meetings and/or travel (payment to institution) from Gedeon Richter. These companies had no role in the study. The remaining authors have no conflicts or interests to declare.

**REGISTRATION NUMBER:**

The study is registered in PROSPERO, ID number CRD42024537107.

WHAT DOES THIS MEAN FOR PATIENTS? This study looks at how many women come back to use their frozen ovarian tissue, which was preserved to protect their fertility before undergoing treatments like chemotherapy that could damage their ability to have children. The study reviewed all the available research on how often women return to use their frozen ovarian tissue by searching medical research databases. The researchers found that the return rate ranged from 0% to 14%, but most of the studies showed that fewer than 5% returned to use their frozen ovarian tissue. The low return rate found in this study raises questions about the actual need of freezing ovarian tissue to protect fertility. However, because some women may wait several years before using their frozen ovarian tissue, the results could be affected by not having a long enough follow-up period. Furthermore, most studies did not account for the fact that some of the women or children who had their tissue frozen were still too young or had not yet shown an interest in having children by the time the study ended. The low return rate found in this study emphasizes the need for future studies with longer follow-up to better understand who actually uses their frozen ovarian tissue. This includes looking at diagnosis and age at the time of freezing, to help prevent women from facing unnecessary, expensive, and potentially risky treatments.

## Introduction

Gonadotoxic treatment for both malignant and benign conditions, including chemotherapy, radiation therapy, and hematopoietic stem cell transplantation, can damage the ovarian reserve and possibly result in premature ovarian insufficiency (POI) and infertility (Meirow, 2000; Overbeek *et al.*, 2017; van Dorp *et al.*, 2018; Su *et al.*, 2025). While the incidence of cancer among women of reproductive age is increasing, the overall survival rate after cancer is also rising (Nordcan 2.0, 2024). Many women postpone childbearing, and as a result, may not have started or completed their family by the time they receive gonadotoxic treatment (Eurostat, 2025). The risk of treatment-induced infertility is a major concern among these women (Partridge *et al.*, 2004; Peate *et al.*, 2009; Howard-Anderson *et al.*, 2012; Bentsen *et al.*, 2023). The area of reproductive medicine focusing on fertility preservation (FP) prior to gonadotoxic treatment has evolved rapidly during the last few decades, and the number of clinics that guide women and offer FP treatments, including ovarian tissue cryopreservation (OTC), has increased worldwide (Donnez and Dolmans, 2017; Anderson *et al.*, 2020).

Donnez *et al.* (2004) reported the first live birth after auto-transplantation of cryopreserved ovarian tissue, and OTC has been recognized as an option for FP by the American Society for Reproductive Medicine (ASRM) since 2019 (Practice Committee of the American Society for Reproductive Medicine, 2019). OTC typically involves the removal of an entire ovary or ovarian cortical biopsies under general anesthesia usually via laparoscopic surgery. The excised ovary is then dissected into cortical strips which are cryopreserved (Rosendahl *et al.*, 2011). Slow freezing remains the current standard technique for OTC (Anderson *et al.*, 2017), whereas vitrification is emerging as an alternative approach (Sänger *et al.*, 2024). Autologous ovarian tissue transplantation (AOTT) can be performed in women exhibiting signs of POI following completion of gonadotoxic treatment, with the aim of restoring endocrine function and/or achieving pregnancy, either naturally or through ovarian stimulation and IVF. Live birth rates following AOTT have been reported ranging from 25% to 41% (Colmorn *et al.*, 2022; Lotz *et al.*, 2022; Fraison *et al.*, 2023) with no increased risk of perinatal complications compared with the general population except for preeclampsia (Erden *et al.*, 2024). Even though OTC in many clinics is not the first choice of FP (Rodriguez-Wallberg *et al.*, 2019), it is still considered relevant in premenarchal girls, post-menarche girls considered too young for ovarian stimulation and egg retrieval, and in women who are experiencing time constraints due to the urgency of initiating chemotherapy, and therefore do not have the time to go through ovarian stimulation. Although OTC is generally considered safe with little risk of complications (Beckmann *et al.*, 2018), surgery still carries risks that may delay the initiation of life-saving chemotherapy. Furthermore, a proportion of the women may never develop POI following gonadotoxic treatment, and therefore, the use of costly and potentially risky treatments as a precautionary measure should be carefully evaluated, particularly when it involves removing healthy ovarian tissue, which could potentially reduce the ovarian reserve further. These factors underscore the importance of evaluating return rates.

A considerable number of years have passed since the first ovarian tissue was cryopreserved, and many studies are now able to report long-term follow-up data. This enables evaluation of FP treatment options and the populations to whom they are offered. The aim of this systematic review was to assess the return rates for the use of ovarian tissue cryopreserved as FP prior to gonadotoxic treatment.

## Methods

The study was registered in PROSPERO (ID number CRD42024537107). We made no amendment after registration except from using an alternative risk of bias evaluation tool (described below). We used the Preferred Reporting Items for Systematic Reviews and Meta-Analysis (PRISMA) guidelines in the development of the systematic review (Page *et al.*, 2021).

### Information sources and eligibility criteria

We searched PubMed (MEDLINE), EMBASE, and the Cochrane Library using medical subject headings (MeSH), Emtree Terms, and text words. Retrospective and prospective cohort studies, case-control studies, and randomized controlled trials that reported the proportion of women who returned for AOTT out of the total number who underwent OTC prior to gonadotoxic treatment were included. Studies that examined only the return rate for oocyte and/or embryo cryopreservation, and studies that investigated the outcomes from OTC without reporting the proportion of women who returned for AOTT were excluded. Furthermore, we excluded studies in languages other than English, abstracts without full text, letters to the editor, and case series involving fewer than 25 females undergoing OTC. Systematic reviews were screened for relevant references but not included in data extraction.

### Search strategy

We developed the search strategy by combining search terms relating to FP, OTC and return rates in collaboration with a health science librarian, who specializes in systematic review searching. No filters or limits were used. The last search was run on 11 March 2025. We manually screened reference lists of selected studies for additional relevant articles. The following search strategy was conducted for the PubMed Search:

(((“Fertility Preservation”[Mesh]) OR (“fertility preser*”[Title/Abstract])) AND (((ovar*[Title/Abstract]) AND (cryopreserv*[Title/Abstract])) OR ((“Cryopreservation”[Mesh]) AND (“Ovary”[Mesh])))) AND (((((((outcome*[Title/Abstract]) OR (“return rate*”[Title/Abstract])) OR (retransplant*[Title/Abstract])) OR (“autologous transplant*”[Title/Abstract])) OR (autotransplant*[Title/Abstract])) OR (transplant*[Title/Abstract])) OR (“Transplantation”[Mesh]))

For additional strategies, see Supplementary Table S1.

### Study selection

**Figure 1.**
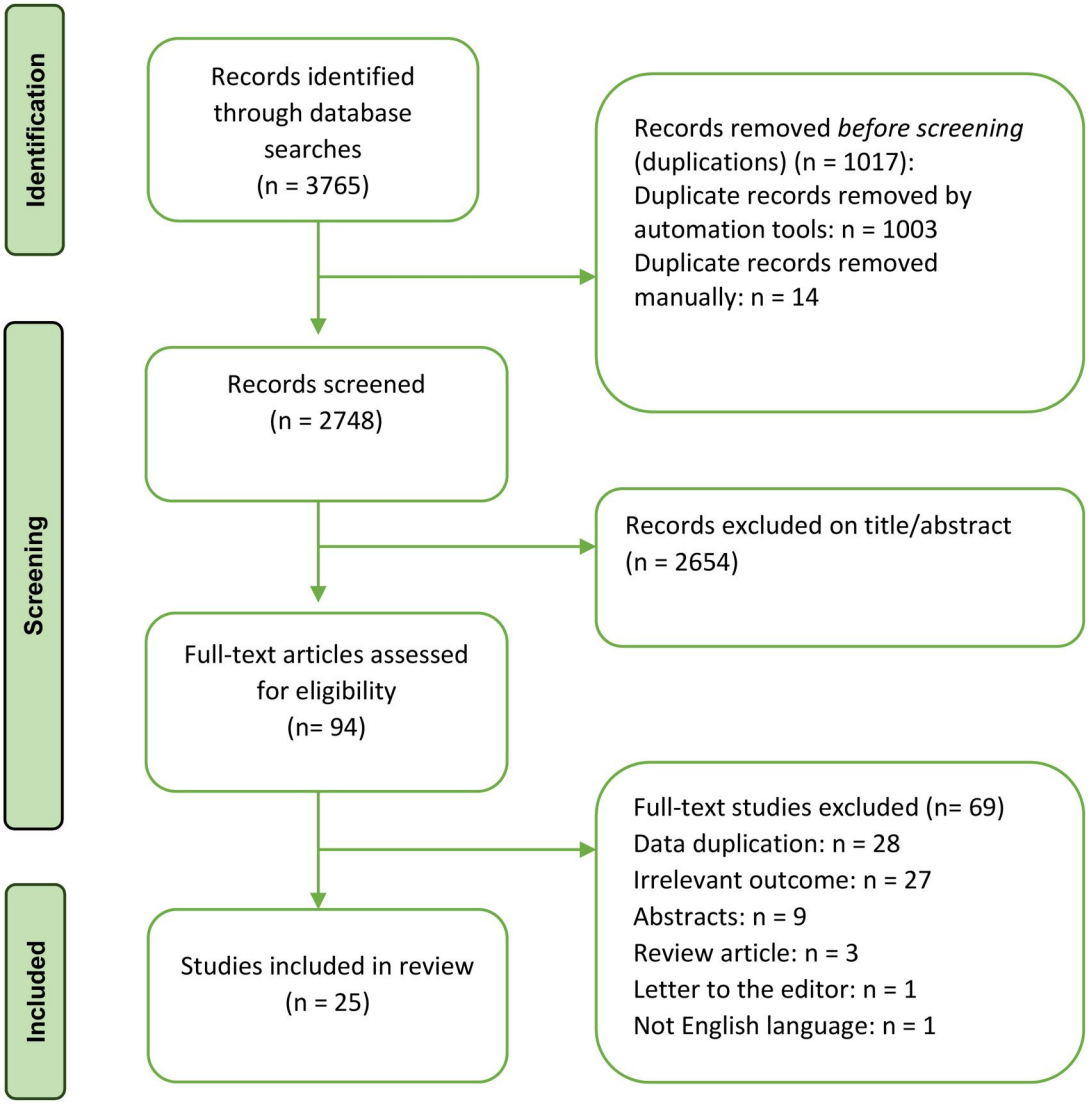
**Flow-chart for the study inclusion in a systematic review on the return rates for the use of cryopreserved ovarian tissue**.

We imported the search results to Covidence (Veritas Health Innovation, Melbourne, Australia) to facilitate deselection of data duplicates, and the collaboration between reviewers regarding the screening process. Four reviewers took part in the screening process (A.M.Y., L.B.C., A.S.J., and K.T.M.). Each study was evaluated independently by two reviewers who were blinded to each other’s choices. Studies were screened according to relevance regarding the topic, content, and inclusion/exclusion criteria. In case of disagreement between reviewers in any point during the screening process, consensus was reached either through discussion or by consulting a third reviewer. In cases where more than one study was published using the same population or dataset, the most recent publication was included, and the other study/studies were excluded at the full-text screening stage due to ‘data duplication’. The study selection process is depicted in the PRISMA flowchart (Fig. 1).

### Data extraction

A data extraction template was developed and agreed on by all reviewers. Data were extracted by one reviewer (A.M.Y.) and validated by a second reviewer (K.T.M.). From each included study, we extracted the following information: author, year of publication, title, country of publication, study design, and outcome details including the number of females receiving OTC prior to gonadotoxic treatment and the number of women returning for AOTT. When available, we also extracted the following data: age at OTC and AOTT, indication for OTC and AOTT, time interval from OTC to AOTT, follow-up time, proportion of participants aged ≥18 years at the end of follow-up (EOF), and the number of women lost to follow-up and mortality during the study period.

### Data synthesis

**Figure 2.**
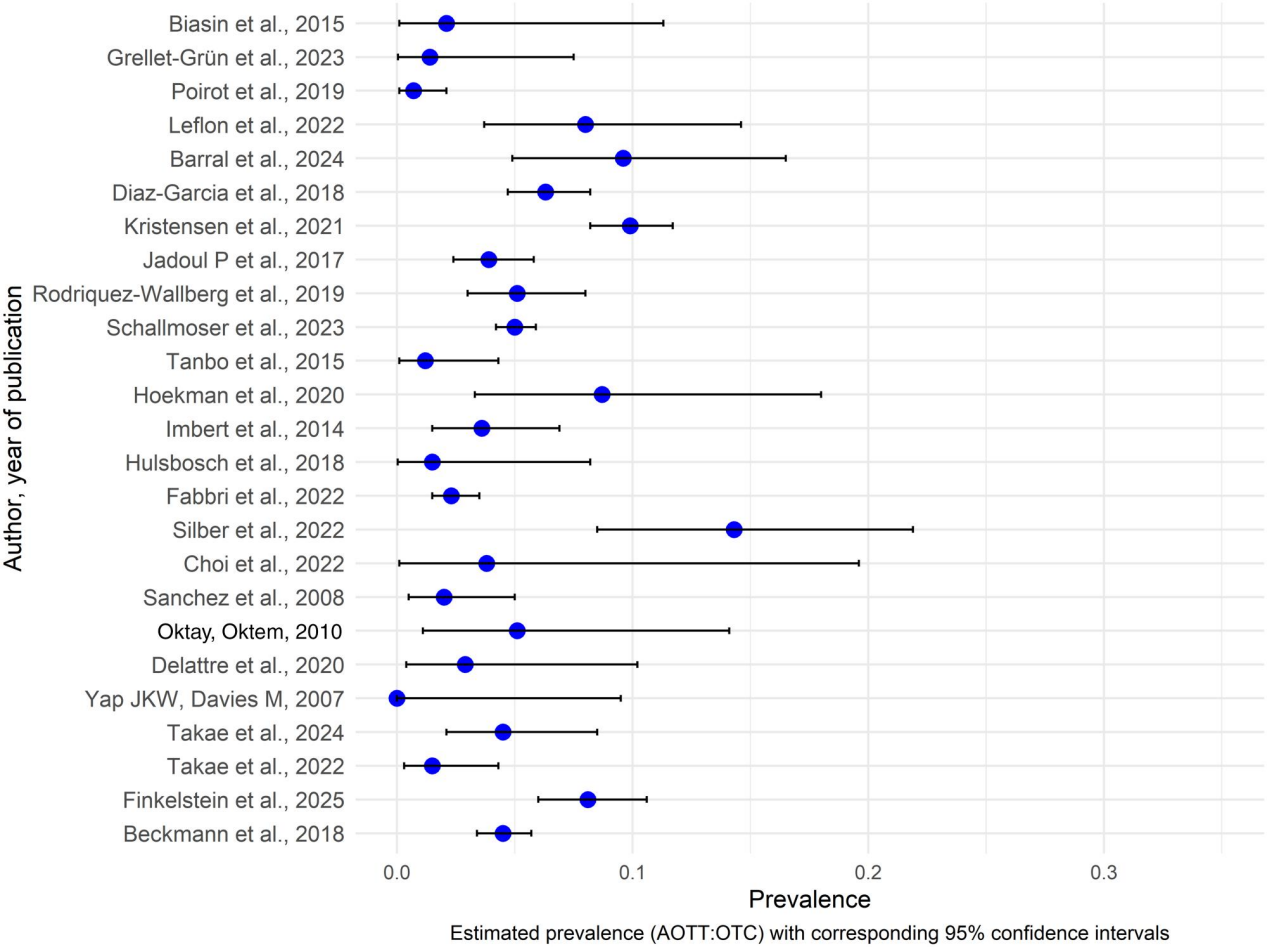
**Forest plot of the return rates for the use of ovarian tissue cryopreserved prior to gonadotoxic treatment**. Prevalence of women returning for autotransplantation out of those who had their tissue frozen with corresponding 95% CIs.

We performed a synthesis by summarizing the results through text and tables following the Synthesis Without Meta-Analysis (SWiM) in systematic reviews: reporting guideline (Campbell *et al.*, 2020). The outcome metric was the proportion of women who underwent AOTT among those who had their tissue cryopreserved (AOTT:OTC), presented as percentage. We generated a Forest plot (Fig. 2) using the statistical software R (version 4.4.1; R Foundation for Statistical Computing, Vienna, Austria), including all eligible studies, illustrating the prevalence of the utilization rate with 95% CIs. Due to differences between children and adults (indications for OTC, risk of POI following gonadotoxic treatment, and the number of participants who were old enough to undergo AOTT or expressing a desire for pregnancy at the EOF), studies were grouped by age at OTC (children, adults, or both) within Table 1, Supplementary Table S2, and Fig. 2. Due to substantial heterogeneity in study characteristics (age at OTC and AOTT, follow-up time, indications for OTC) we did not perform a meta-analysis, as it would not yield a meaningful summary estimate.

**Table 1.** Study characteristics and results: return rates for the use of ovarian tissue cryopreserved prior to gonadotoxic treatment.

Author, year, country, title	Study design, cohort	Follow-up (FU) time	Indications for fertility preservation	Review outcomes	Comments
Biasin *et al.*, 2015 Italy ‘Ovarian tissue cryopreservation in girls undergoing hematopoietic stem cell transplant: Experience of a single center’	**Design:** cohort study **Time period of OTC:** August 2000–September 2013 **Cohort:** n=47 **Age at OTC:** median age 13 years (range 2.7–20.3 years) Pre-pubertal: n=24 (51%) Pubertal: n=23 (49%) **Time from OTC to AOTT:** not specified **Proportion of participants aged ≥18 years at EOF:** Median age at last follow-up was 18.6 years (range 5.46–29.36 years) **Lost to FU and mortality during the study period:** deceased: n=7	**FU Time:** Median FU time 6.54 years (range 0.30–13.68 years)	**Reason for OTC:** HSCT (Diamond-Blackfan anemia (n=1), Ewing sarcoma (n=3), immunodeficiency (n=2), AML (n=11), ALL (n=14), CML (n=5), non-Hodgkin lymphoma (n=2), MDS (n=2), thalassemia (n=7)). **Reason for AOTT:** not specified	**OTC:** n=47 **AOTT:** n=1 (2%)	
Grellet-Grün *et al.*, 2023 France ‘A 16-year biocentric retrospective analysis of ovarian tissue cryopreservation in pediatric units: indications, results, and outcome’	**Design:** retrospective cohort study **Time period of OTC:** January 2004–May 2020 **Cohort:** n=72 **Age at OTC:** mean age 9.3 years (range 0.2–17 years) Pre-pubertal: n=51 Post-pubertal: n=21 **Time from OTC to AOTT:** 14 years **Proportion of participants aged ≥18 years at EOF:** 14:72 ≥18 years (19%), 5:72 ≥23 years (7%) **Lost to FU and mortality during the study period:** deceased: n=15	**FU Time:** Median post-harvest FU time was 92 months (1–188 months)	**Reason for OTC:** malignant disease: n=51 (70.8%) (malignant hematological diseases n=25 (35%), solid malignant tumors n=26 (36%)). nonmalignant disease: 21 (29.1%) (Hemoglobinopathies n=15 (21%), other non-malignant diseases n=6 (8%)). **Reason for AOTT:** not specified	**OTC:** n=72 **AOTT:** n=1 (1.4%)	
Poirot *et al.*, 2019 France ‘Ovarian tissue cryopreservation for fertility preservation in 418 girls and adolescents up to 15 years of age facing highly gonadotoxic treatment. Twenty years of experience at a single center’	**Design:** retrospective cohort study **Time period of OTC:** April 1998–December 2018 **Cohort:** n=418 **Age at OTC:** ≤15 years median age 6.9 years (range 0.3–15 years) <10 years: n=278 (66.5%) <5 years: n=150 (35.9%) **Time from OTC to OTT:** not specified **Proportion of participants aged ≥18 years at EOF:** n=149 **Lost to FU and mortality during the study period:** deceased: n=84 (20.1%)	**FU Time:** not specified (within the timeframe of the study)	**Reason for OTC:** malignant diseases: n=313 (74.8%) (hematological malignancy n=97 (23.2%), solid tumor n=218 (51.7%)) non-malignant diseases: n=105 (25.2%) (hemoglobinopathies n=71 (68.9%)). **Reason for AOTT:** restore endocrine function: n=1 restore fertility: n=2	**OTC:** n=418 **AOTT:** n=3 (0.7%)	Return rate for only those alive and 18 years or older at EOF: 3:149=2.2%
Leflon *et al.*, 2022 France ‘Experience, and gynecological and reproductive health follow-up of young adult women who have undergone ovarian tissue cryopreservation’	**Design:** retrospective observational cohort study **Time period of OTC:** 2004–2018 **Cohort:** n=113 **Age at OTC:** ≥18 years, mean age 29.5±4.8 years (range 18–37 years) **Time from OTC to AOTT:** not specified **Proportion of participants aged ≥18 years at EOF:** 100% **Lost to FU and mortality during the study period:** deceased: n=14 (12%)	**FU Time:** Average interval between OTC and the medical FU consultation was 88.9 months (range 20.2–187.9 months)	**Reason for OTC:** hematological pathologies: n=49 (43%) (lymphoma n=36 (32%), leukemia n=6 (5%), non-malignant hematological disease requiring HSCT n=7 (6%)), breast cancer: n=34 (30%), gastrointestinal malignancies: n=8 (7%), sarcoma: n=5 (4%), gynecological malignancies n=4 (4%), larynx cancer: n=1 (0.9%), non-malignant disease requiring chemotherapy or radiotherapy: n=5 (4%), benign and borderline ovarian pathologies: n=7 (6.2%)) **Reason for AOTT:** not specified	**OTC:** n=113 **AOTT:** n=9 (8.0%)	11 patients (9.7%) requested AOTT
Barral *et al.*, 2024 Spain ‘Current status of fertility preservation in a Spanish tertiary public hospital: multidisciplinary approach and experience in over 1500 patients’	**Design:** retrospective cohort study **Time period of OTC:** 2006–2022 **Cohort:** n=703 (OTC: 115, OC: 304, GnRH agonist: 175, fertility sparing treatments for gynecological cancer: 109) **Age at OTC:** mean age of the entire cohort: 31.8 years (range 16–40 years). **Time from OTC to OTT:** mean 6.17 years **Proportion of participants aged ≥18 years at EOF:** not specified **Lost to FU and mortality during the study period:** deceased: 15:115 (13%)	**FU Time:** not specified (within the timeframe if the study)	**Reason for OTC (diagnoses):** breast cancer: 81% **Reason for AOTT:** not specified	**OTC:** n=115 **AOTT:** n=11 (9.5%)	
Diaz-Garcia *et al.*, 2018 Spain ‘Oocyte vitrification versus ovarian cortex transplantation in fertility preservation for adult women undergoing gonadotoxic treatments: a prospective cohort study’	**Design:** prospective cohort study **Time period of OTC:** 2005–2015 **Cohort:** n=800 OTC (+1024 OV) **Age at OTC:** 28.2 years (±7.3 years) **Time from OTC to AOTT:** mean storage time 5.5 years **Proportion of participants aged ≥18 years at EOF:** 100% **Lost to FU and mortality during the study period:** not specified	**FU Time:** Median follow-up time for the **e**ntire cohort (both OTC and OV) was 4.89 years (range 0.39–10.97 years)	**Reason for OTC:** breast cancer: n=431 (53.9%), Hodgkin lymphoma: n=159 (19.9%), non-Hodgkin lymphoma: n=24 (3%), gynecological malignancies: n=25 (3.1%), sarcoma: n=52 (6.5%), leukemia: n=54 (6.8%), autoimmune disease: n=20 (2.5%), other solid organ tumors: n=35 (4.4%) **Reason for OTT:** seeking pregnancy: n=44 (88%) endocrine purposes: n=6 (12%)	**OTC:** n=800 **AOTT:** n=50 (6.2%)	
Kristensen *et al.*, 2021 Denmark ‘Use of cryopreserved ovarian tissue in the Danish fertility preservation cohort’	**Design:** retrospective cohort study **Time period of OTC:** 1999–2020 **Cohort:** n=1186 **Age at OTC entire cohort:** mean age 25.1 years (±9 years), ranging from 4 months to 43 years. ≤18 years: n=242 (21%) 19–34 years: n=833 (70%) ≥35: n=111 (9%) Subgroup 1: 24.6 years (±9 years) **Age at AOTT:** not specified **Time from OTC to AOTT: m**ean storage time 4.3 years **Proportion of participants aged ≥18 years at EOF:** not specified **Lost to FU and mortality during the study period:** deceased total number: n=142 (12%) deceased subgroup 1: n=135 (18%) donated tissue for science: n=72 (6%)	**FU time:** Mean FU time was 8 years (entire cohort). Mean FU time in subgroup 1 was 10.9 years, minimum 5 years	**Reason for OTC:** malignant indications: breast cancer (38%), malignant hematologic diseases (25%) (lymphoma 18%, leukemia 7%), sarcoma (8%), gynecological malignancies (8%), neurological malignancies (5%), gastrointestinal malignancies (3%), other malignant diseases (e.g. kidney, nasopharyngeal cancer (1%)) benign indications, 12%: benign hematological diseases (5%), systemic diseases (rheumatologic and autoimmune disease (3%)), genetic diseases (2%). **Reason for AOTT:** achieve pregnancy: n=106 restore hormone production: n=10 induce puberty: n=1	*Entire cohort:* **OTC:** n=1186 **AOTT:** n=117 (10%) *Subgroup 1:* **OTC:** n=759 **AOTT:** n=104 (14%) *Subgroup 2* **OTC:** n=554 **AOTT:** n=103 (19%)	Subgroup 1: >5 years FU Subgroup 2: alive and aged >24 years in July 2020
Jadoul *et al.*, 2017 Belgium ‘Efficacy of ovarian tissue cryopreservation for fertility preservation: lessons learned from 545 cases’	**Design:** retrospective cohort study **Time period of OTC:** 1997–2013 **Cohort:** n=545 **Age at OTC:** ≤35 years. mean age 22.3±8.8 years (range 6 months–39 years) ≤18 years: n=157 (29%) prepubertal: n=80 (15%) >18 to ≤35: n=388 (71%) **Age at OTT:** not specified **Time from OTC to AOTT:** not specified **Proportion of participants aged ≥18 years at EOF:** not specified **Lost to FU and mortality during the study period:** deceased: n=54 (10%)	**FU Time: M**ean FU time was 7.6 years (±3.5 years)	**Reason for OTC:** lymphomas (23%), leukemia (9%), benign hematological pathologies requiring bone marrow transplantation (3%), breast cancer (17%), sarcoma (9%), gynecological malignancies (6%), neurological malignancies (5%), gastrointestinal malignancies (3%), systemic diseases requiring chemotherapy (2%), benign and borderline ovarian pathologies (17.5%), genetic risk of POI (turner syndrome, family history of early menopause or galactosemia) (3.5%) **Reason for AOTT:** not specified	**OTC:** n=545 **AOTT:** n=21 (3.9%)	24 (4.4%) patients requested AOTT
Rodriguez-Wallberg *et al.*, 2019 Sweden ‘A prospective study of women and girls undergoing fertility preservation due to oncologic and non-oncologic indications in Sweden-Trends in patients’ choices and benefit of the chosen methods after long-term follow up’	**Design:** prospective cohort study **Time period of OTC:** 1998–2018 **Cohort of women receiving FP counselling:** n=1254 adults: n=1076 children: n=178 **Age at OTC:** adult women, n=221: mean age 28.1 years (range 18–39 years) post-pubertal teenagers, n=66: mean age 15.6 years (range 14–17 years) pre-pubertal, n=48: mean age 11.2 years (range 3–13 years) **Time from OTC to AOTT:** not specified **Proportion of participants aged ≥18 years at EOF:** not specified **Lost to FU and mortality during the study period:** deceased: n=97:1254 (7.7%) emigrated: n=18:1254 (1.4%)	**FU Time:** Mean FU time (for the entire cohort of women receiving FP) was 6.1 years (± 4.8 years) (range 0–19 years)	**Reason for FP counselling (entire cohort of women receiving FP counselling):** malignant diseases: n=852 benign diseases: n=402 **Reason for AOTT:** not specified	**OTC:** n=335 **AOTT:** 5%	Utilization rate was calculated as the number of patients who returned for AOTT out of all patients aged 18–40 years who had undergone FP, and were alive and living in Sweden at least 1 year following FP
Schallmoser *et al.*, 2023 Germany ‘Cryostorage of human ovarian tissue: evaluating the storage and disposal pattern over a 22-year period in 2475 patients’	**Design:** retrospective cohort study **Time period of OTC:** 2000–2021 **Cohort:** n=2475 **Age at OTC:** median age 28 years (min–max 0–44) **Time from OTC to AOTT:** not specified **Proportion of participants aged ≥18 years at EOF:** not specified **Lost to FU and mortality during the study period:** deceased: n=152	**FU Time:** FU-time was depicted as storage duration (years): ≥5 years active storage: n=661 (median storage duration 7.4 years) (range 5–16.3 years) ≥10 years active storage: n=148 (median storage duration 11.9 years) (range 10–16.3 years) Ended storage: n=1155 (median storage duration 3.8 years) (range 0–19.1 years)	**Reason for OTC:** hematological malignancies: n=60 (8.4%), brain and nervous system: n=72 (2.9%), sarcoma: n=146 (5.9%), gynecological tumors: n=162 (6.5%, breast cancer: n=1108 (44.8%), lymphoma: n=555 (22.4%), Others: n=207 (8.4%) non-specified: 165 (6.7%)) **Reason for OTT:** not specified	**OTC:** n=2475 **AOTT:** n=124 (5%)	Of the 124 AOTT 29 (1.17%) were performed on site and 95 (3.83%) were outsourced for scheduled transplantation at other sites.
Tanbo *et al.*, 2015 Norway ‘Autotransplantation of cryopreserved ovarian tissue after treatment for malignant disease—the first Norwegian results’	**Design:** retrospective cohort study **Time period of OTC:** 2004–2014 **Cohort:** n=164 **Age at OTC:** ≤35 years **Time from OTC to AOTT:** not specified **Proportion of participants aged ≥18 years at EOF:** not specified **Lost to FU and mortality during the study period:** deceased: n=15	**FU Time:** not specified (within the timeframe of the study)	**Reason for OTC:** breast cancer: 40%, lymphomas: 25%, sarcomas: 15%, other malignant or benign conditions: 20%. **Reason for AOTT:** not specified	**OTC:** n=164 **AOTT:** n=2 (1.2%)	6:162 patients (3.6%) requested AOTT (1 patient conceived spontaneously immediately before AOTT; 1 patient was diagnosed with BRCA1 and BRCA2 gene mutations, and had a family history of several BRCA related cancers, and was therefore advised against AOTT; 1 patient had leukemia-specific gene re-arrangements (IgH and TCRD/A) in the cryopreserved ovarian tissue; 1 patient treated for diffuse natural killer cell lymphoma was under assessment for AOTT at the time of the study)
Hoekman *et al.*, 2020 Netherlands ‘Ovarian tissue cryopreservation: Low usage rates and high live-birth rate after transplantation’	**Design:** retrospective cohort study **Time period of OTC:** 2002–2015 **Cohort:** n=69 **Age at OTC:** ≤36 years mean age 24 years (range 10.2–35.7 years) <18 years: n=19 ≥18: n=50 **Time from OTC to OTT:** not specified **Proportion of participants aged ≥18 years at EOF:** not specified **Lost to FU and mortality during the study period:** deceased: n=12 (17.4%)	**FU Time:** FU ended in October 2018. Mean FU time was 77.4 months (range 4–183 months)	**Reason for OTC:** malignant diagnoses: n=56 (81.2%) (breast cancer n=25 (36.2%), other malignant diseases n=31 (44.9%)) benign indications: n=13 (18.9%) **Reason for AOTT:** not specified	**OTC:** n=69 **AOTT:** n=6 (8.7%)	
Imbert *et al.*, 2014 Belgium ‘Safety and usefulness of cryopreservation of ovarian tissue to preserve fertility: a 12-year retrospective analysis’	**Design:** retrospective cohort study **Time period of OTC:** March 1999–June 2011 **Cohort:** n=225 **Age at OTC:** ≤36 years aged 0.8–17 years: n=45 (20%) aged ≥18 years: n=180 (80%) **Time from OTC to OTT:** not specified **Proportion of participants aged ≥18 years at EOF:** not specified **Lost to FU and mortality during the study period:** deceased: n=28	**FU-Time:** not specified (within the timeframe of the study)	**Reason for OTC:** prepubertal (n=27): hematological benign diseases (58%), lymphoma (4%), leukemia (27%), solid tumor (7%), immunological diseases (4%) post-pubertal (n=198): breast cancer (43%), lymphoma (22%), leukemia (6%), ovarian borderline tumor (8%), solid tumor (7%), pelvic tumor (8%), immunological disease (5%), hematological benign disease (1%) **Reason for AOTT:** not specified	**OTC:** n=225 **AOTT:** n=8 (3.6%)	
Hulsbosch *et al.*, 2018 Belgium ‘A real-life Analysis of Reproductive Outcome after Fertility Preservation in Female Cancer Patients’	**Design:** retrospective cohort study **Time period of OTC:** January 1999–December 2011 **Cohort:** n=159 **Age at OTC:** post-menarche women, mean age 23 years (range 11–37 years) **Time from OTC to AOTT:** not specified **Proportion of participants aged ≥18 years at EOF:** not specified **Lost to FU and mortality during the study period:** deceased: n=15 (23%)	**FU Time:** ≥3 years after the end of primary anticancer treatment, mean FU time: 61.5 months	**Reason for OTC:** based on the entire cohort (66 had OTC, the rest had GnRH agonist treatment only): hematological cancer: n=77 (48.4%), gynecological cancer: n=48 (30.2%), soft tissue sarcoma: n=16 (10.1%), gastro intestinal cancer: n=6 (3.8%), miscellaneous: n=12 (7.5%) (neuroblastoma, medulloblastoma, thymoma, adrenocortical carcinoma, glioma, mesothelioma) **Reason for AOTT:** not specified	**OTC:** n=66 **AOTT:** n=1 (1.5%)	
Fabbri *et al*., 2022 Italy ‘Ovarian tissue cryopreservation and transplantation: 20 years experience in Bologna University’	**Design:** retrospective cohort study **Time period of OTC:** January 2002–January 2022 **Cohort:** n=1026 **Age at OTC:** 2–38 years ≤17 years: 238 (23.2%) (group 1) 18–38 years: 788 (76.8%) (group 2) mean age ±SD: group 1: 12.9 ± 4.14 group 2: 28.0 ± 5.7 **Time from OTC to AOTT:** mean storage time 7.48 years ±3.5 years (range 2–17 years) **Proportion of participants aged ≥18 years at EOF:** not specified **Lost to FU and mortality during the study period:** deceased: n=68 (6.6%) (group 1: n=27, group 2: n=41).	**FU Time:** not specified (within the timeframe of the study)	**Reason for OTC:** malignant diseases: n=930 (91%) (lymphomas: n=419, leukemias: n=30, myelodysplasia: n=13, breast cancers: n=260, sarcomas: n=101, neurological malignancies: n=39, gastrointestinal malignancies: n=26, gynecological malignancies: n=22, Wilms tumor: n=9, others: n=11) non-malignant diseases: n=96 (9%) (genetic diseases: n=52, autoimmune diseases: n=17, others: n=27) **Reason for OTT:** restore-and or improve ovarian function and seek pregnancy: n=20 restore steroidogenesis: n=4	**OTC:** 1026 **AOTT:** 24 (2.3%)	
Silber *et al.*, 2022 USA ‘*In vitro* maturation and transplantation of cryopreserved ovary tissue: understanding ovarian longevity’	**Design:** retrospective cohort study **Time period of OTC:** 1997–2020 **Cohort:** n=119 **Age at OTC:** 1–42 years **Time from OTC to AOTT:** not specified **Proportion of participants aged ≥18 years at EOF:** not specified **Lost to FU and mortality during the study period:** not specified	**FU Time:** not specified (within the timeframe of the study)	**Reason for OTC:** cancer: n=85 premature ovarian failure: n=8 social reasons: n=13 others (turner syndrome, endometriosis, MS, aplastic anemia, massive ovarian bilateral teratoma or a daughter born with no ovary) **Reason for AOTT:** not specified	**OTC:** n=119 **OTT:** n=17 (14%)	The 17 recipients were between 18 and 31 years at OTC (median age of 24 years)
Choi *et al.*, 2022 Korea ‘The experience of Fertility Preservation in a Single Tertiary Center in Korea’	**Design:** retrospective cohort study **Time period of OTC:** 2010–October 2021 **Cohort:** n=26 **Age at OTC:** 11–41 years **Time from OTC to AOTT:** not specified **Proportion of participants aged ≥18 years at EOF:** not specified **Lost to FU and mortality during the study period:** not specified	**FU Time:** Mean duration of the cryopreserved ovarian tissue was 4.98 (±2.85) years and the longest was stored for 12.5 years	**Reason for OTC:** breast cancer n=2 (7.7%), hematologic cancer n=5 (19.2%), gynecologic cancer n=9 (34.6%), gastrointestinal cancer n=1 (3.9%), other malignancy n=5 (19.2%), benign ovarian cyst n=1 (3.9%), impending POI n=3 (11.5%) **Reason for OTT:** not specified	**OTC:** n=26 **OTT:** n=1 (3.8%)	
Sánchez *et al.*, 2008 Valencia ‘The Valencia Programme for Fertility Preservation’	**Design:** cohort study **Time period of OTC:** not specified **Cohort:** n=200 **Age at OTC:** mean age 28.2 years (range 11–39 years) **Time from OTC to OTT:** not specified **Proportion of participants aged ≥18 years at EOF:** not specified **Lost to FU and mortality during the study period:** not specified	**FU Time:** not specified (within the timeframe of the study)	**Reason for OTC:** breast cancer: 55%, HD: 25% other malignant or non-malignant diseases: 20% (colorectal carcinoma, sarcoma, glomerulonephritis, cancer, lupus, other) **Reason for AOTT:** not specified	**OTC:** n=200 **AOTT:** 4 (2%)	
Oktay and Oktem, 2010 USA ‘Ovarian cryopreservation and transplantation for fertility preservation for medical indications: report of an ongoing experience’	**Design:** prospective longitudinal analysis **Time period of OTC:** May 1997–March 2008 **Cohort:** n=59 **Age at OTC:** mean age 26.7 years (±1.2 years) (range 4–44 years) 0–18 years: 19% 19–29 years: 42% 30–39 years: 31% 40–44 years: 8% **Time from OTC to AOTT:** 6 months–2 years **Proportion of participants aged ≥18 years at EOF:** pre-pubertal at FU: n=3 **Lost to FU and mortality during the study period:** deceased: n=4 (8%)	**FU Time:** Median length of storage was 3.06 (±0.2) years (0.05–10.5 years)	**Reason for OTC:** breast cancer: 22%, Hodgkin disease: 21%, non-Hodgkin lymphoma: 10.2%, acute myelitic leukemia: 10.2%, acute lymphocytic leukemia: 5.1%, early ovarian carcinoma: 5.1%, endometrial carcinoma: 3.4%, cervical carcinoma: 3.4%, others: 20.3% (aplastic anemia, diamond-blackfan syndrome, myelodysplasia, thalassemia major, hemophagocytic lymphohistiocytosis, lupus nephritis, ependymoma, synovial sarcoma, mosaic karyotype, vanishing bone disease, endometriosis, oophorectomy for dermoid cysts) **Reason for AOTT:** not specified	**OTC:** n=59 **AOTT:** n=3 (5%)	
Delattre *et al.*, 2020 Belgium ‘Combining fertility preservation procedures to spread the eggs across different baskets: a feasibility study’	**Design:** retrospective observational study **Time period of OTC:** January 2012–December 2018 **Cohort:** n=207 **Age at OTC:** prepubertal: n=13 OTC (n=4) mean age ±SD: 5.5 years (±7.1) OTC + OTO–IVM (n=9) mean age ±SD: 5.1 years (± 3.6) post-pubertal: n=55 OTC + OTO–IVM (n=24) mean age ±SD: 27.9 years (±6.6) OPU–IVM + OTC + OTO–IVM (n=17) mean age ±SD: 25.9 years (± 4.8) OTC + OTO–IVM COS (n=13) mean age ± SD: 26.2 years (± 4.3) **Time from OTC to AOTT:** not specified **Proportion of participants aged ≥18 years at EOF:** not specified **Lost to FU and mortality during the study period:** not specified	**FU Time:** not specified (within the time frame of the study)	**Reason for OTC based on the entire cohort of the study:** breast cancer n=95, hematological cancer n=43, gynecological cancer n=31, neurological cancer n=18, colorectal cancer n=6, sarcoma n=8, other types, n=6. **Reason for AOTT:** not specified	**OTC:** n=68 **AOTT:** n=2 (2.9%)	
Yap and Davies, 2007 UK ‘Fertility preservation in female cancer survivors’	**Design:** retrospective cohort study **Time period of OTC:** March 1995–March 2005 **Cohort:** 37 **Age at OTC:** mean age 27.0 years (range 2–42) **Time from OTC to AOTT:** not specified **Proportion of participants aged ≥18 years at EOF:** not specified **Lost to FU and mortality during the study period:** deceased: n=4	**FU Time:** not specified (within the timeframe of the study)	**Reason for OTC (diagnoses):** breast cancer: n=14 (37.8%), gynecological cancer: n=9 (24.3%), other solid: n=2 (5.4%), hematological cancer: n=6 (16.2%), solid cancer child: =1 (2.7%) **Reason for AOTT:** not specified	**OTC:** n=37 **AOTT:** n=0 (0%)	
Takae *et al.*, 2024 Japan ‘Survey on the implementation status and reproductive outcomes of oocyte and ovarian tissue cryopreservation in Japan: Historical comparison with nationwide surveys’	**Design:** mailed-in questionnaire survey **Time period of OTC:** December 2016–December 2020 **Cohort:** n=198 **Age at OTC (for the ones receiving OTT):** mean age 36±3.9 years **Time from OTC to AOTT:** Average 5±1.6 years (median, 5 years) **Proportion of participants aged ≥18 years at EOF:** not specified **Lost to FU and mortality during the study period:** not specified	**FU Time:** not specified (within the timeframe of the study)	**Reason for OTC:** not specified **Reason for AOTT:** not specified	**OTC:** n=198 **AOTT:** n=9 (4.5%)	Survey targeting 51 facilities, response rate of 43:51 (84.3%). OTC was performed in 18 facilities
Takae *et al.*, 2022 Japan ‘A practical survery of fertility preservation treatments in the startup phase in Japan’	**Design:** mailed-in questionnaire survey **Time period of OTC:** January 2006–November 2016 **Cohort:** n=201 **Age at OTC:** mean age 29.7 ± 8.3 years (range 5–46 years). <10: n=3 11–15: n=23 16–20: n=17 21–25: n=26 26–30: n=37 31–35: n=57 36–40: n=33 41–45: n=4 46–50: n=1 <15 years: 12.9% **Time from OTC to AOTT:** not specified **Proportion of participants aged ≥18 years at EOF:** not specified **Lost to FU and mortality during the study period:** deceased: n=14 (7%)	**FU Time:** not specified (within the timeframe of the study)	**Reason for OTC (diagnoses):** breast cancer: n=89, malignant lymphoma: n=19, leukemia: n=18, bone and soft tissue tumor: n=8, brain tumor: n=7, ovarian tumor: n=7, uterine cervical cancer: n=7, autoimmune disease: n=7, myelodysplastic syndrome: n=5, ovarian cancer: n=5, ovarian tumor: n=5, endometrial cancer: n=5, ewing sarcoma: n=4, aplastic anemia: n=3, other hematological diseases: n=2, kidney cancer: n=2, colon cancer: n=2, others: n=7 **Reason for AOTT:** not specified	**OTC:** n=201 **AOTT:** n=3 (1.4%)	100% survey response rate from 30 facilities that performed OTC
Finkelstein *et al.*, 2025 Australia ‘Pregnancy outcomes following ovarian tissue cryopreservation: an Australian cohort study’	**Design:** retrospective cohort study **Time period of OTC:** 1995–2022 **Cohort:** n=593 **Age at OTC:** mean age 27.2 years (SD 7.3) (range 9–44 years) **Time from OTC to AOTT:** 8.8 years (mean age at OTT 36.0 years) **Proportion of participants aged ≥18 years at EOF:** not specified **Lost to FU and mortality during the study period:** deceased: n=107 (18.0%)	**FU Time:** not specified (within the timeframe of the study)	**Reason for OTC:** transplant: solid tumor cancer 24:48 (50.0%), hematological cancer 13:48 (27.1%), autoimmune disorder 6:48 (12.5%), benign gynecological disorder 2:48 (4.2%), other benign disease 2:48 (4.2%), other cancer 1:48 (2.1%) non-transplant: solid tumor cancer 275:545 (50.5%), hematological cancer 171:545 (31.4%), autoimmune disorder 24:545 (4.4%), benign gynecological disorder 21:545 (3.9%), other benign disease 21:545 (3.9%), other cancer 21:545 (3.9%), genetic disorder 8:545 (1.5%), donor 3:545 (0.6%), not reported 1:545 (0.2%) **Reason for AOTT:** not specified	**OTC:** n=593 **AOTT:** n=48 (8.1%)	
Beckmann *et al.*, 2018 FertiProtekt (Germany, Austria, Switzerland) ‘Fertility protection: complications of surgery and results of removal and transplantation of ovarian tissue’	**Design:** retrospective cohort study **Time period of OTC:** 2007–2016 **Cohort:** n=1302 **Reason for AOTT:** not specified **Age at OTC:** not specified **Time from OTC to AOTT:** average storage time: 3 years **Proportion of participants aged ≥18 years at EOF:** not specified **Lost to FU and mortality during the study period:** not specified	**FU Time:** not specified (within the timeframe of the study)	**Reason for OTC:** breast cancer: n=552 (42.4%), Hodgkin’s disease: n=282 (21.7%), sarcoma: n=65 (5%), non-Hodgkin’s lymphoma: n=44 (3.4%), leukemia: n=40 (3.1%), cervical carcinoma: n=28 (2.2%), ovarian germ cell tumor: n=24 (1.8%), central nervous system tumors: n=16 (1.2%), rectal carcinoma: n=15 (1.2%), ovarian borderline tumor: n=14 (1.1%), ovarian carcinoma: n=14 (1.1%), anal carcinoma: n=5 (0.4%), vulvar carcinoma: n=4 (0.3%), gastric carcinoma: n=4 (0.3%), colon carcinoma: n=3 (0.2%), peritoneal carcinoma: n=3 (0.2%), endometrial carcinoma: n=2 (0.2%), renal cell carcinoma: n=2 (0.2%), chordoma: n=2 (0.2%), melanoma: n=1 (0.1%), carcinoma of unknown primary: n=1 (0.1%), synovial carcinoma: n=1 (0.1%), cholangiocarcinoma: n=1 (0.1%), carcinoma of the tongue: n=1 (0.1%), hydatiform mole: n=1 (0.1%), pharyngeal carcinoma: n=1 (0.1%), unavailable details on diagnoses: n=89 (6.8%) Benign disease: n=85 (6.5%)	**OTC:** n=1302 **AOTT:** n=58 (4.5%)	

OTC, ovarian tissue cryopreservation; AOTT, autologous ovarian tissue transplantation; EOF, end of follow-up; FU, follow-up; FP, fertility preservation; HSCT, hematopoietic stem cell transplantation; AML, acute myeloid leukemia; ALL, acute lymphoblastic leukemia; CML, chronic myeloid leukemia; MDS, myelodysplastic syndrome; OV, oocyte vitrification; OTO–IVM, ovarian tissue oocyte–*in vitro* maturation; OPU–IVM, oocyte pick-up–*in vitro* maturation; COS, controlled ovarian stimulation.

### Risk of bias assessment and quality assessment

We assessed risk of bias for each included study using the Joanna Briggs Institute (JBI) Cohort Checklist. Although most of the included studies primarily investigated outcomes related to FP treatment success (e.g. pregnancy rate), secondary data on the return rate for the use of cryopreserved ovarian tissue were extracted for the purpose of this review. Risk of bias for this outcome was therefore assessed using the JBI Critical Appraisal Checklist for Cohort Studies, which is appropriate for evaluating studies reporting descriptive data on prevalence or return rates (Joanna Briggs Institute, 2020). The overall certainty of evidence was assessed using Grading of Recommendations Assessment, Development, and Evaluation (GRADE), considering risk of bias, inconsistency, indirectness, imprecision, and publication bias.

## Results

After screening 2748 studies on title abstract and 94 studies on full text, 25 studies were found eligible for inclusion. The study selection process is depicted in the PRISMA flow chart (Fig. 1). All studies were cohort studies. Supplementary Tables S2 and S3 present the Risk of Bias Assessment according to JBI Critical Appraisal Checklist for Cohort Studies and quality assessment according to GRADE, respectively. Unspecified or insufficient follow-up time was a possible source of bias in all included studies (Supplementary Table S2). Some studies addressed this limitation through sensitivity analyses. For example, Kristensen *et al.* performed sensitivity analysis by stratifying the population by >5 years of follow-up (subgroup 1) and by >5 years of follow-up, alive and aged >24 years (subgroup 2) (Kristensen *et al.*, 2021). The overall quality of the studies in reporting the return rate for the use of cryopreserved ovarian tissue was moderate (Supplementary Table S3).

Table 1 provides an overview of the study characteristics and outcomes across the 25 included studies. The studies were largely heterogeneous regarding cohort size, time period during which OTC was performed, indication for OTC, age at the time of OTC, and follow-up time.

The number of females undergoing OTC varied substantially across the included studies, ranging from 26 participants in the smallest study (Choi *et al.*, 2022), to 2475 in the largest (Schallmoser *et al.*, 2023). Three studies reported cohorts of ≤50 (Yap and Davies, 2007; Biasin *et al.*, 2015; Choi *et al.*, 2022). Five reported cohorts of >50–100 (Oktay and Oktem, 2010; Hulsbosch *et al.*, 2018; Delattre *et al.*, 2020; Hoekman *et al.*, 2020; Grellet-Grün *et al.*, 2023). Six reported cohorts of >100–200 (Sánchez *et al.*, 2008; Tanbo *et al.*, 2015; Leflon *et al.*, 2022; Silber *et al.*, 2022; Barral *et al.*, 2024; Takae *et al.*, 2024). Three studies reported cohorts of >200–400 participants (Imbert *et al.*, 2014; Rodriguez-Wallberg *et al.*, 2019; Takae *et al.*, 2022), and another three reported cohorts of >400–600 (Jadoul *et al.*, 2017; Poirot *et al.*, 2019; Finkelstein *et al.*, 2025). One study reported a cohort of 800 participants (Diaz-Garcia *et al.*, 2018). Additionally, three reported cohorts of >1000–1500 (Beckmann *et al.*, 2018; Kristensen *et al.*, 2021) and one reported a cohort of >2000 (Schallmoser *et al.*, 2023). All OTC procedures were performed between 1999 and 2022. In all studies, OTC was performed for both malignant and benign indications; however, malignant disease made up the more frequent indication for OTC. Fabbri *et al.* reported that among 1026 patients who underwent OTC, 91% (n = 930) did so for malignant indications, whereas 9% (n = 96) did so for non-malignant indications (Fabbri *et al*., 2022), while Beckmann *et al.* reported that only 6.3% of the women had their tissue frozen for benign indications (Beckmann *et al.*, 2018) (Table 1). The age at OTC ranged from 0 to 44 years. Three studies included only pediatric patients (aged ≤20 years at OTC) (Biasin *et al.*, 2015; Poirot *et al.*, 2019; Grellet-Grün *et al.*, 2023), three studies included only adolescent and adult patients (≥16 years) (Diaz-Garcia *et al.*, 2018; Leflon *et al.*, 2022; Barral *et al.*, 2024), 18 studies included both pediatric and adult patients at the time of OTC (Yap and Davies, 2007; Sánchez *et al.*, 2008; Oktay and Oktem, 2010; Imbert *et al.*, 2014; Tanbo *et al.*, 2015; Jadoul *et al.*, 2017; Hulsbosch *et al.*, 2018; Rodriguez-Wallberg *et al.*, 2019; Delattre *et al.*, 2020; Hoekman *et al.*, 2020; Kristensen *et al.*, 2021; Choi *et al.*, 2022; Silber *et al.*, 2022; Takae *et al.*, 2022, 2024; Schallmoser *et al.*, 2023; Finkelstein *et al.*, 2025), and 1 study did not specify the age of the patients at the time of OTC (Beckmann *et al.*, 2018).

In most included studies, the follow-up time was not clearly defined but occurred within the overall time frame of the study (Table 1). For example, an Australian study by Finkelstein *et al.*, which assessed return rates among women who underwent OTC between 1995 and 2022, reported that 48 of 593 women had returned for OTT (8.1%). However, the follow-up duration was not specified (Finkelstein *et al.*, 2025). Except from one study that reported a minimum follow-up duration of ≥3 years after primary cancer treatment (Hulsbosch *et al.*, 2018), none of the other studies reported a defined overall minimum follow-up duration (Table 1). Most studies did not account for patient mortality when calculating overall return rates; however, in one study, the overall return rate was calculated as the proportion of women who returned out of those who were alive (Rodriguez-Wallberg *et al.*, 2019).

The return rate for use of cryopreserved ovarian tissue after completion of gonadotoxic treatment is depicted in the Forest plot (Fig. 2) and ranged from 0% to 14%. Of the 25 studies, 18 reported a return rate of ≤5%, 6 reported a return rate of >5% to ≤10% while only 1 study reported a return rate of 14%.

When stratified by age group at the time of OTC, studies including only pediatric patients reported return rates ranging from 0.7% to 2% (Biasin *et al.*, 2015; Poirot *et al.*, 2019; Grellet-Grün *et al.*, 2023), compared to return rates of 6.2–9.5% (Diaz-Garcia *et al.*, 2018; Leflon *et al.*, 2022; Barral *et al.*, 2024) in studies including only adolescents and adults (≥16 years).

## Discussion

Overall, we found a low return rate of 0–14% after OTC, with most of the studies reporting return rates of ≤5%. In most studies, follow-up occurred within the time frame of the study, with only 8 studies explicitly reporting the follow-up duration, which ranged from 0 to 19.1 years (Oktay and Oktem, 2010; Biasin *et al.*, 2015; Diaz-Garcia *et al.*, 2018; Rodriguez-Wallberg *et al.*, 2019; Hoekman *et al.*, 2020; Leflon *et al.*, 2022; Grellet-Grün *et al.*, 2023; Schallmoser *et al.*, 2023) (Table 1). The reasons for the modest return rates can be manyfold. Key factors influencing the return rate for AOTT include: 1) length of follow-up, 2) need for AOTT (development of POI after OTC), 3) desire to undergo AOTT, and 4) the actual feasibility of AOTT, including mortality among those who had undergone OTC.

### Length of follow-up

Since AOTT may occur several years after OTC, limited follow-up time may bias the results. In all included studies, a subset of participants remained within reproductive age at EOF. Furthermore, most of the studies included both children and adults who underwent OTC and did not exclude individuals younger than 18 years of age at EOF when calculating return rates. Consequently, these analyses may underestimate true utilization rates by not accounting for participants who were not yet eligible for tissue transplantation (e.g. actively undergoing gonadotoxic therapy or in convalescence) or had not reached reproductive age or expressed a desire for pregnancy yet. Consistent with this, Emrich *et al.* (2025) examined storage patterns across age groups at the time of OTC and found that children and adolescents had a significantly higher proportion of active storage beyond 10 years compared with adults.

### Need for AOTT (development of POI)

The risk of POI and infertility following cancer treatment depends, among others, on the type and cumulative dose of chemo and radiation therapy and therefore varies depending on the primary cancer diagnosis (Schüring *et al.*, 2018; Van Den Berg *et al.*, 2018). A substantial proportion of women who underwent OTC had been diagnosed with breast cancer. In the study by Schallmoser *et al*., women diagnosed with breast cancer accounted for 44.8% of patients who underwent OTC (1108 out of 2475), whereas in the study by Diaz-Garcia *et al*., it was 53.9% (431 out of 800) (Diaz-Garcia *et al.*, 2018; Schallmoser *et al.*, 2023). However, many clinics no longer offer OTC as the first choice of FP to women with breast cancer, partly because the likelihood of natural pregnancy after treatment in this patient group is high (Partridge *et al.*, 2023; Magaton *et al.*, 2025), and partly because oocyte or embryo cryopreservation provides additional benefits, such as the possibility of pre-implantation genetic testing (PGT) for individuals carrying BRCA 1/2 gene mutations (Macklon and De Vos, 2024).

In a sub-analysis of a Danish cohort, Kristensen *et al*. reported the use of cryopreserved ovarian tissue among patients who were alive and >24 years in 2020, stratified by diagnosis. They found a lower return rate among women diagnosed with breast cancer (18%) compared to that of women diagnosed with other malignant diseases (gastrointestinal malignancies (27%), gynecological malignancies (22%), sarcoma (21%)) (Kristensen *et al.*, 2021).

Young age at cancer treatment has a mitigating effect on the risk of POI (Schüring *et al.*, 2018; Anderson *et al.*, 2022) and therefore, utilization rate for AOTT may be higher among women diagnosed at a more advanced age. Kristensen et al. (2021) assessed the impact of age at the time of OTC on subsequent return rates. They found that women who underwent OTC aged ≥30 years had a return rate of 15%, nearly twice that of those aged 18–29 years (8%) (Kristensen *et al.*, 2021). In many facilities, OTC is recommended only for women aged <35 years (Anderson *et al.*, 2020), due to unfavorable results in those >35 years, and the lower risk of infertility after gonadotoxic treatment in younger patients may therefore also contribute to the overall low return rate. This may also partially explain the low return rates reported in studies that include only pediatric patients.

### Desire to undergo AOTT

Patients undergoing OTC for FP prior to gonadotoxic treatment may experience a change in their desire to have children following cancer treatment. For many cancer survivors, fear of disease recurrence is a significant concern and the possibility of bringing a child into the world who might lose a parent at an early age may also play an important role in their decision-making regarding attempts to conceive (Liu *et al.*, 2025). A Belgian study by Hulsbosch *et al*. showed that among women in remission who had undergone OTC±GnRH agonist (n = 39), 46% (n = 18) expressed a desire for pregnancy, while 54% (n = 21) did not, after a minimum of 3-year follow-up (Hulsbosch *et al.*, 2018). A Danish study by Macklon *et al*. found that among those who requested disposal of their cryopreserved ovarian tissue after an initial period of at least 5 years (17%), 19% did so because they decided not to have children (Macklon *et al.*, 2014). Furthermore, following a prolonged treatment period, often including both surgical and medical interventions, patients may be reluctant to undergo additional surgical procedures required for transplantation of the cryopreserved tissue. Schallmoser *et al*. assessed the reason for ending storage among 224 patients who had undergone OTC for FP. They showed that 25.9% ended storage due to a lack of desire to have children, while 3.1% cited fear of future surgery as a reason. Additional reasons for discontinuing storage included pregnancy (natural or after IVF treatment), cancer recurrence, high storage costs, or patient death (Schallmoser *et al.*, 2023).

### Actual feasibility of AOTT, including mortality

The low return rate, when reported without accounting for mortality, may be further influenced by the fact that some of the diagnoses associated with a high risk of infertility also carry a high mortality risk. Macklon (2019) found a mortality rate of 13% in a Danish cohort of 927 girls and women who underwent OTC with the most deaths observed in the group with upper gastro-intestinal cancers and sarcomas. Furthermore, oocyte and embryo cryopreservation have partly replaced OTC as preferred FP method in recent times (Anderson *et al.*, 2020). Consequently, OTC is increasingly offered only to women with limited time before starting chemotherapy, often due to advanced cancer progression. Therefore, women undergoing OTC for FP, particularly in more recent times, may have a worse prognosis, potentially reducing the number of women who survive long enough to utilize the cryopreserved tissue and thereby contributing to the lower observed return rates.

### Heterogeneity between studies

The return rate for the use of cryopreserved ovarian tissue varied substantially across included studies, possibly explained by the heterogeneity between studies including age at OTC, follow-up time, indications for OTC, and country of FP. Stratified by age at OTC, the utilization rate among patients who had their tissue frozen as children (range 0.7–2%) was lower compared to the rate among those who had their tissue frozen as adolescents and adults (range 6.2–9.5%). As return for AOTT may occur several years after OTC, the lower return rate observed among children may be partly explained by the fact that they have not yet reached reproductive age or have not yet expressed desire for childbearing by the end of the follow-up period. Further studies with extended follow-up duration are needed.

Furthermore, in a Belgian study by Delattre *et al.,* OTC was performed in addition to either controlled ovarian stimulation (COS), oocytes retrieved from ovarian tissue *ex vivo* (OTO-IVM), or transvaginal retrieval of oocytes for IVM. Consequently, the return rate for utilization of the cryopreserved ovarian tissue may be lower in these patients as they would likely choose to use their cryopreserved oocytes or embryos first in order to avoid another surgical procedure (Delattre *et al.*, 2020). This may also apply to some of the other included studies, as it is unclear whether they exclusively had OTC performed or if other FP methods were used simultaneously.

Overall, studies have reported higher return rates for the use of cryopreserved oocytes, embryos, and semen compared to OTC. A review by Wnuk *et al*. (2023) found that return rates for the use of oocytes, embryos, and semen cryopreserved prior to gonadotoxic treatment ranged from 3.1% to 8.7%, 9% to 22.4%, and 2.6% to 21%, respectively.

Variations among countries concerning patient-borne cost of the treatment may result in differences between populations receiving OTC and the length of storage. In countries where patients must cover the costs themselves, OTC is more likely to be pursued by women with a clear intention to undergo AOTT, if needed. In contrast, in countries where the procedure is offered to patients free of charge, a greater proportion of women may choose to undergo it as a precautionary measure, even though they may never use it. This may partly explain the high return rate in the study by Silber *et al.* (2022).

Cultural and religious variations between countries regarding whether women are reluctant to undergo AOTT if they are unmarried may also contribute to variations in return rates between countries. Studies have shown that cancer survivors are less likely to marry, compared to the general population, and they may therefore, in some countries, have a reduced likelihood of becoming mothers (Syse, 2008; Kirchhoff *et al.*, 2012).

Furthermore, eligibility criteria for OTC may vary between countries, influenced by disease severity and associated mortality risk. This is particularly important, as most studies do not account for mortality when reporting return rates.

### Limitations

Although this systematic review is up to date and methodologically robust, with broad search criteria, it has several limitations. Many of the included studies either lack follow-up information or fail to specify a minimum follow-up duration. The studies are also heterogeneous in their populations, particularly regarding patient age. Moreover, standardized definitions of ‘return’ are absent. These limitations may contribute to underreporting and bias. Return rate was not the primary outcome of most of the included studies, and therefore, there is a risk of underreporting or inconsistent outcome ascertainment. This may influence the reliability of the prevalence estimates reported.

## Conclusion

Despite the common practice of offering OTC as FP, the low utilization rates highlight the need for careful consideration to avoid subjecting women to potentially unnecessary and costly treatment. The procedure diminishes the ovarian reserve and, like all surgical interventions, carries risks of complications such as infection and bleeding, that could, if severe enough, delay subsequent cancer treatment. Reassuringly, however, studies have shown a high satisfaction rate and a low complication rate (Beckmann *et al.*, 2018). On the other hand, the importance of the hope that OTC represents for the patients during a very difficult time should not be underestimated either (Bach *et al.*, 2020; Bentsen *et al.*, 2023). The overall modest return rates for the use of ovarian tissue cryopreserved prior to gonadotoxic treatment found in this study emphasize the importance of future studies with longer follow-up time to assess patterns of tissue utilization in relation to diagnosis, treatment protocol, and age thereby setting criteria in the selection of patients who would actually benefit from OTC.

## Supplementary Material

hoaf068_Supplementary_Data

## Data Availability

The data underlying this article will be shared on reasonable request to the corresponding author.
