# Supplementary material for: Return rates for the use of ovarian tissue cryopreserved prior to gonadotoxic treatment as fertility preservation: a systematic review
Source: Hum Reprod Open. 2025 Oct 28;2025(4):hoaf068. doi: 10.1093/hropen/hoaf068 (PMC12638063; doi:10.1093/hropen/hoaf068)
Supplement: hoaf068_Supplementary_Data [file hoaf068_supplementary_data.zip › Supplementary Table S3_GRADE.docx]

**Supplementary Table S3** Overall certainty of evidence assessed using GRADE: Return rates for the use of ovarian tissue cryopreserved prior to gonadotoxic treatment as fertility preservation: a systematic review

| Domain | Assessment | GRADE |
| --- | --- | --- |
| Risk of bias | In all 25 included studies, outcome and exposure were measured in a valid and reliable way. In all studies the outcome may occur several years after exposure, and therefore insufficient follow-up time may bias the results. | Moderate |
| Inconsistency | Inconsistency regarding clinical heterogeneity: Differences between study populations in terms of age (children/adults), reason for OTC, and follow-up time. | Moderate |
| Indirectness | Direct evidence for target population and outcome measurements. | High |
| Imprecision | Large cumulative sample size and total number of events | High |
| Publication bias | No suspected publication bias | High |
| Overall GRADE |  | Moderate |
