# Supplementary material for: Return rates for the use of ovarian tissue cryopreserved prior to gonadotoxic treatment as fertility preservation: a systematic review
Source: Hum Reprod Open. 2025 Oct 28;2025(4):hoaf068. doi: 10.1093/hropen/hoaf068 (PMC12638063; doi:10.1093/hropen/hoaf068)
Supplement: hoaf068_Supplementary_Data [file hoaf068_supplementary_data.zip › Supplementory Table S1_litterature search.docx]

**Supplementary Table S1** Search strategy: Return rates for the use of ovarian tissue cryopreserved prior to gonadotoxic treatment as fertility preservation: a systematic review

|  | **Block #1:** Fertility preservation | **Block #2:** Ovarian tissue cryopreservation | **Block #3:** Return rates |
| --- | --- | --- | --- |
| **Text words** | “Fertility preser*” | Cryopreserv*  AND  Ovar* | Transplant*  OR  autotransplant*  OR  ”Autologous transplant*”  OR  Retransplant*  OR  ”return rate*”  OR  Outcome* |
| **Controlled subject headings** | Fertility preservation | Cryopreservation  AND  ovary | Transplatation |

| **Database/date of last search** | **Search strategy** | **Results** |
| --- | --- | --- |
| **PubMed**  11/3-2025 | **#1 ("Fertility Preservation"[Mesh]) OR ("fertility preser*"[Title/Abstract])**  **#2 ((ovar*[Title/Abstract]) AND (cryopreserv*[Title/Abstract])) OR (("Cryopreservation"[Mesh]) AND ("Ovary"[Mesh]))**  #3 **((((((outcome*[Title/Abstract]) OR ("return rate*"[Title/Abstract])) OR (retransplant*[Title/Abstract])) OR ("autologous transplant*"[Title/Abstract])) OR (autotransplant*[Title/Abstract])) OR (transplant*[Title/Abstract])) OR ("Transplantation"[Mesh])**  **#1 AND #2 AND #3** | 1593 |
| **Embase**  11/3-2025 | #1 exp fertility preservation/  OR  "fertility preser* ".ab,kf,ti.  #2 "cryopreserv*".ab,kf,ti. AND "ovar*".ab,kf,ti.  OR  exp cryopreservation/ AND exp ovary/  #3 (transplant* or autotransplant* or "autologous transplant*" or retransplant* or outcome* or "return rate*").ab,kf,ti.  OR  exp transplantation/  #1 AND #2 AND #3 | 2151 |
| **Cochrane Library**  11/3-2025 | #1 ("fertility preservation"):ti,ab,kw (Word variations have been searched)  OR  MeSH descriptor: [Fertility Preservation] explode all trees  #2 ("cryopreservation"):ti,ab,kw (Word variations have been searched) AND (ovary):ti,ab,kw (Word variations have been searched)  OR  MeSH descriptor: [Cryopreservation] explode all trees AND MeSH descriptor: [Ovary] explode all trees  #3 (transplantation):ti,ab,kw (Word variations have been searched) OR ("autotransplantation"):ti,ab,kw (Word variations have been searched) OR ("autologous transplantation"):ti,ab,kw (Word variations have been searched) OR ("retransplantation"):ti,ab,kw (Word variations have been searched) OR ("return rate"):ti,ab,kw (Word variations have been searched) OR (outcome):ti,ab,kw (Word variations have been searched)  OR  MeSH descriptor: [Transplantation] explode all trees  #1 AND #2 AND #3 | 21 |
