# Supplementary material for: Return rates for the use of ovarian tissue cryopreserved prior to gonadotoxic treatment as fertility preservation: a systematic review
Source: Hum Reprod Open. 2025 Oct 28;2025(4):hoaf068. doi: 10.1093/hropen/hoaf068 (PMC12638063; doi:10.1093/hropen/hoaf068)
Supplement: hoaf068_Supplementary_Data [file hoaf068_supplementary_data.zip › Supplementary_Table_S2_risk_of_bias_assessment.docx]

**Supplementary Table S2:** Risk of bias assessment according to Joanna Briggs Institute (JBI) Critical Appraisal Checklist for Cohort Studies: Return rates for the use of ovarian tissue cryopreserved prior to gonadotoxic treatment as fertility preservation: as systematic review

| **Author, year, country** | **Study quality (JBI critical appraisal checklist for cohort studies)** | **Judgment (Yes/No/Unclear/not applicable)** | **Comments** |
| --- | --- | --- | --- |
| Biasin et al.  2015  Italy | 1. Were the two groups similar and recruited from the same population? | Yes  No  Unclear  Not applicable | 1.-2. No exposure group comparison  4.-5. Confounding factors were not considered as the study is descriptive and does not compare exposure groups.  8. median FU time 6.54 years (range 0.30-13.68 years). Median age at last follow-up was 18.6 years (range 5.46-29.36 years). A large proportion of participants were not old enough to actually use the tissue at the end of follow-up. Furthermore, return for OTT may occur several years after OTC and insufficient time may therefore bias the results.  10. No sensitivity analysis was reported for incomplete follow-up/deceased patients. |
|  | 2. Were the exposures measured similarly to assign people to both exposed and unexposed groups? | Yes  No  Unclear  Not applicable |  |
|  | 3. Was the exposure measured in a valid and reliable way? | Yes  No  Unclear  Not applicable |  |
|  | 4. Were confounding factors identified? | Yes  No  Unclear  Not applicable |  |
|  | 5. Were strategies to deal with confounding factors stated? | Yes  No  Unclear  Not applicable |  |
|  | 6. Were the groups/participants free of the outcome at the start of the study (or at the moment of exposure)? | Yes  No  Unclear  Not applicable |  |
|  | 7. Were the outcomes measured in a valid and reliable way? | Yes  No  Unclear  Not applicable |  |
|  | 8. Was the follow up time reported and sufficient to be long enough for outcomes to occur? | Yes  No  Unclear  Not applicable |  |
|  | 9. Was follow up complete, and if not, were the reasons for loss to follow up described and explored? | Yes  No  Unclear  Not applicable |  |
|  | 10. Were strategies to address incomplete follow-up utilized? | Yes  No  Unclear  Not applicable |  |
|  | 11. Was appropriate statistical analysis used? | Yes  No  Unclear  Not applicable |  |
| Grellet-Grün et al.  2023  France | 1. Were the two groups similar and recruited from the same population? | Yes  No  Unclear  Not applicable | 1.-2. No exposure group comparison  4.-5. Confounding factors were not considered as the study is descriptive and does not compare exposure groups.  8. median post-harvest FU time was 92 months (1-188). Return for OTT may occur several years after OTC and insufficient time may therefore bias the results.  10. No sensitivity analysis was reported for incomplete follow-up/deceased patients. |
|  | 2. Were the exposures measured similarly to assign people to both exposed and unexposed groups? | Yes  No  Unclear  Not applicable |  |
|  | 3. Was the exposure measured in a valid and reliable way? | Yes  No  Unclear  Not applicable |  |
|  | 4. Were confounding factors identified? | Yes  No  Unclear  Not applicable |  |
|  | 5. Were strategies to deal with confounding factors stated? | Yes  No  Unclear  Not applicable |  |
|  | 6. Were the groups/participants free of the outcome at the start of the study (or at the moment of exposure)? | Yes  No  Unclear  Not applicable |  |
|  | 7. Were the outcomes measured in a valid and reliable way? | Yes  No  Unclear  Not applicable |  |
|  | 8. Was the follow up time reported and sufficient to be long enough for outcomes to occur? | Yes  No  Unclear  Not applicable |  |
|  | 9. Was follow up complete, and if not, were the reasons for loss to follow up described and explored? | Yes  No  Unclear  Not applicable |  |
|  | 10. Were strategies to address incomplete follow-up utilized? | Yes  No  Unclear  Not applicable |  |
|  | 11. Was appropriate statistical analysis used? | Yes  No  Unclear  Not applicable |  |
| Poirot et al.  2019  France | 1. Were the two groups similar and recruited from the same population? | Yes  No  Unclear  Not applicable | 1.-2. No exposure group comparison  4.-5. Confounding factors were not considered as the study is descriptive and does not compare exposure groups.  8. follow-up time was not specified (no information of minimum FU), however the study describes 20 years’ experience of OTC. Return for OTT may occur several years after OTC and insufficient time may therefore bias the results.  10. Return rate for only those alive and 18 years or older at EOF was reported. |
|  | 2. Were the exposures measured similarly to assign people to both exposed and unexposed groups? | Yes  No  Unclear  Not applicable |  |
|  | 3. Was the exposure measured in a valid and reliable way? | Yes  No  Unclear  Not applicable |  |
|  | 4. Were confounding factors identified? | Yes  No  Unclear  Not applicable |  |
|  | 5. Were strategies to deal with confounding factors stated? | Yes  No  Unclear  Not applicable |  |
|  | 6. Were the groups/participants free of the outcome at the start of the study (or at the moment of exposure)? | Yes  No  Unclear  Not applicable |  |
|  | 7. Were the outcomes measured in a valid and reliable way? | Yes  No  Unclear  Not applicable |  |
|  | 8. Was the follow up time reported and sufficient to be long enough for outcomes to occur? | Yes  No  Unclear  Not applicable |  |
|  | 9. Was follow up complete, and if not, were the reasons for loss to follow up described and explored? | Yes  No  Unclear  Not applicable |  |
|  | 10. Were strategies to address incomplete follow-up utilized? | Yes  No  Unclear  Not applicable |  |
|  | 11. Was appropriate statistical analysis used? | Yes  No  Unclear  Not applicable |  |
| Leflon et al.  2022  France | 1. Were the two groups similar and recruited from the same population? | Yes  No  Unclear  Not applicable | 1.-2. No exposure group comparison  4.-5. Confounding factors were not considered as the study is descriptive and does not compare exposure groups.  8. Return for OTT may occur several years after OTC and insufficient time may therefore bias the results.  9. Although the study included a patient-reported follow-up questionnaire, only registry-based return data on OTT were used in this review. Therefore, the completeness and validity of follow-up were assessed based on availability of return outcome data.  10. No sensitivity analysis was reported for incomplete follow-up/deceased patients. |
|  | 2. Were the exposures measured similarly to assign people to both exposed and unexposed groups? | Yes  No  Unclear  Not applicable |  |
|  | 3. Was the exposure measured in a valid and reliable way? | Yes  No  Unclear  Not applicable |  |
|  | 4. Were confounding factors identified? | Yes  No  Unclear  Not applicable |  |
|  | 5. Were strategies to deal with confounding factors stated? | Yes  No  Unclear  Not applicable |  |
|  | 6. Were the groups/participants free of the outcome at the start of the study (or at the moment of exposure)? | Yes  No  Unclear  Not applicable |  |
|  | 7. Were the outcomes measured in a valid and reliable way? | Yes  No  Unclear  Not applicable |  |
|  | 8. Was the follow up time reported and sufficient to be long enough for outcomes to occur? | Yes  No  Unclear  Not applicable |  |
|  | 9. Was follow up complete, and if not, were the reasons for loss to follow up described and explored? | Yes  No  Unclear  Not applicable |  |
|  | 10. Were strategies to address incomplete follow-up utilized? | Yes  No  Unclear  Not applicable |  |
|  | 11. Was appropriate statistical analysis used? | Yes  No  Unclear  Not applicable |  |
| Barral et al.  2024  Spain | 1. Were the two groups similar and recruited from the same population? | Yes  No  Unclear  Not applicable | 1. And 2. Although the original study included both OTC, OC, EC and GnRH agonist, only data regarding OTC were extracted for this review. No comparison of exposures for the purpose of this review was performed.  4. and 5. Confounding factors were not considered as the study is descriptive and does not compare exposure groups.  8. Follow-up time not specified. Return for OTT may occur several years after OTC and insufficient time may therefore bias the results.  10. No sensitivity analysis was reported for incomplete follow-up/deceased patients. |
|  | 2. Were the exposures measured similarly to assign people to both exposed and unexposed groups? | Yes  No  Unclear  Not applicable |  |
|  | 3. Was the exposure measured in a valid and reliable way? | Yes  No  Unclear  Not applicable |  |
|  | 4. Were confounding factors identified? | Yes  No  Unclear  Not applicable |  |
|  | 5. Were strategies to deal with confounding factors stated? | Yes  No  Unclear  Not applicable |  |
|  | 6. Were the groups/participants free of the outcome at the start of the study (or at the moment of exposure)? | Yes  No  Unclear  Not applicable |  |
|  | 7. Were the outcomes measured in a valid and reliable way? | Yes  No  Unclear  Not applicable |  |
|  | 8. Was the follow up time reported and sufficient to be long enough for outcomes to occur? | Yes  No  Unclear  Not applicable |  |
|  | 9. Was follow up complete, and if not, were the reasons for loss to follow up described and explored? | Yes  No  Unclear  Not applicable |  |
|  | 10. Were strategies to address incomplete follow-up utilized? | Yes  No  Unclear  Not applicable |  |
|  | 11. Was appropriate statistical analysis used? | Yes  No  Unclear  Not applicable |  |
| Diaz-Garcia et al.  2018  Spain | 1. Were the two groups similar and recruited from the same population? | Yes  No  Unclear  Not applicable | 1. And 2. Although the original study included both oocyte vitrification and OTC, only data regarding OTC were extracted for this review. No comparison of exposures for the purpose of this review was performed.  4. and 5. Confounding factors were not considered as the study is descriptive and does not compare exposure groups.  8. follow ranged from 0.39-10.97 years, however return for OTT may occur several years after OTC and insufficient time may therefore bias the results.  9-10. There were no patients lost to follow-up, however the number of deceased patients was not specified. |
|  | 2. Were the exposures measured similarly to assign people to both exposed and unexposed groups? | Yes  No  Unclear  Not applicable |  |
|  | 3. Was the exposure measured in a valid and reliable way? | Yes  No  Unclear  Not applicable |  |
|  | 4. Were confounding factors identified? | Yes  No  Unclear  Not applicable |  |
|  | 5. Were strategies to deal with confounding factors stated? | Yes  No  Unclear  Not applicable |  |
|  | 6. Were the groups/participants free of the outcome at the start of the study (or at the moment of exposure)? | Yes  No  Unclear  Not applicable |  |
|  | 7. Were the outcomes measured in a valid and reliable way? | Yes  No  Unclear  Not applicable |  |
|  | 8. Was the follow up time reported and sufficient to be long enough for outcomes to occur? | Yes  No  Unclear  Not applicable |  |
|  | 9. Was follow up complete, and if not, were the reasons for loss to follow up described and explored? | Yes  No  Unclear  Not applicable |  |
|  | 10. Were strategies to address incomplete follow-up utilized? | Yes  No  Unclear  Not applicable |  |
|  | 11. Was appropriate statistical analysis used? | Yes  No  Unclear  Not applicable |  |
| Kristensen et al.  2021  Denmark | 9. Was follow up complete, and if not, were the reasons for loss to follow up described and explored? | Yes  No  Unclear  Not applicable | 1. and 2. No exposure group comparison  4. and 5. Confounding factors were not considered as the study is descriptive and does not compare exposure groups.  8. Minimum follow up duration for the entire cohort not specified. Return for OTT may occur several years after OTC and insufficient time may therefore bias the results.  10. Sub analysis were made excluding deceased patients |
|  | 10. Were strategies to address incomplete follow-up utilized? | Yes  No  Unclear  Not applicable |  |
|  | 11. Was appropriate statistical analysis used? | Yes  No  Unclear  Not applicable |  |
|  | 4. Were confounding factors identified? | Yes  No  Unclear  Not applicable |  |
|  | 5. Were strategies to deal with confounding factors stated? | Yes  No  Unclear  Not applicable |  |
|  | 6. Were the groups/participants free of the outcome at the start of the study (or at the moment of exposure)? | Yes  No  Unclear  Not applicable |  |
|  | 7. Were the outcomes measured in a valid and reliable way? | Yes  No  Unclear  Not applicable |  |
|  | 8. Was the follow up time reported and sufficient to be long enough for outcomes to occur? | Yes  No  Unclear  Not applicable |  |
|  | 9. Was follow up complete, and if not, were the reasons for loss to follow up described and explored? | Yes  No  Unclear  Not applicable |  |
|  | 10. Were strategies to address incomplete follow-up utilized? | Yes  No  Unclear  Not applicable |  |
|  | 11. Was appropriate statistical analysis used? | Yes  No  Unclear  Not applicable |  |
| Jadoul et al.  2017  Belgium | 1. Were the two groups similar and recruited from the same population? | Yes  No  Unclear  Not applicable | 1. and 2. No exposure group comparison  4. and 5. Confounding factors were not considered as the study is descriptive and does not compare exposure groups.  8. Minimum follow up duration for the entire cohort not specified. Return for OTT may occur several years after OTC and insufficient time may therefore bias the results.  9. Although the study included a patient-reported follow-up questionnaire, only registry-based return data on OTT were used in this review. Therefore assessment was based solely on the latter.  10. No sensitivity analysis was reported for incomplete follow-up/deceased patients. |
|  | 2. Were the exposures measured similarly to assign people to both exposed and unexposed groups? | Yes  No  Unclear  Not applicable |  |
|  | 3. Was the exposure measured in a valid and reliable way? | Yes  No  Unclear  Not applicable |  |
|  | 4. Were confounding factors identified? | Yes  No  Unclear  Not applicable |  |
|  | 5. Were strategies to deal with confounding factors stated? | Yes  No  Unclear  Not applicable |  |
|  | 6. Were the groups/participants free of the outcome at the start of the study (or at the moment of exposure)? | Yes  No  Unclear  Not applicable |  |
|  | 7. Were the outcomes measured in a valid and reliable way? | Yes  No  Unclear  Not applicable |  |
|  | 8. Was the follow up time reported and sufficient to be long enough for outcomes to occur? | Yes  No  Unclear  Not applicable |  |
|  | 9. Was follow up complete, and if not, were the reasons for loss to follow up described and explored? | Yes  No  Unclear  Not applicable |  |
|  | 10. Were strategies to address incomplete follow-up utilized? | Yes  No  Unclear  Not applicable |  |
|  | 11. Was appropriate statistical analysis used? | Yes  No  Unclear  Not applicable |  |
| Rodriguez-Wallberg et al.  2019  Sweden | 1. Were the two groups similar and recruited from the same population? | Yes  No  Unclear  Not applicable | 1. And 2. Although the original study included multiple fertility preservation strategies, only data regarding to OTC were extracted for this review. No comparison of exposures for the purpose of this review was performed; therefore, this domain is not applicable.  4. and 5. Confounding factors were not considered as the outcome for the purpose of this review (OTT) is descriptive and does not compare exposure groups.  8. Minimum FU time was 1 year, however return for OTT may occur several years after OTC and insufficient time may therefore bias the results.  10. Utilization rate was calculated as the number of patients who returned for OTT out of all patients aged 18-40 years who had undergone FP, and were alive and living in Sweden at least 1 year following FP. |
|  | 2. Were the exposures measured similarly to assign people to both exposed and unexposed groups? | Yes  No  Unclear  Not applicable |  |
|  | 3. Was the exposure measured in a valid and reliable way? | Yes  No  Unclear  Not applicable |  |
|  | 4. Were confounding factors identified? | Yes  No  Unclear  Not applicable |  |
|  | 5. Were strategies to deal with confounding factors stated? | Yes  No  Unclear  Not applicable |  |
|  | 6. Were the groups/participants free of the outcome at the start of the study (or at the moment of exposure)? | Yes  No  Unclear  Not applicable |  |
|  | 7. Were the outcomes measured in a valid and reliable way? | Yes  No  Unclear  Not applicable |  |
|  | 8. Was the follow up time reported and sufficient to be long enough for outcomes to occur? | Yes  No  Unclear  Not applicable |  |
|  | 9. Was follow up complete, and if not, were the reasons for loss to follow up described and explored? | Yes  No  Unclear  Not applicable |  |
|  | 10. Were strategies to address incomplete follow-up utilized? | Yes  No  Unclear  Not applicable |  |
|  | 11. Was appropriate statistical analysis used? | Yes  No  Unclear  Not applicable |  |
| Schallmoser et al.  2023  Germany | 1. Were the two groups similar and recruited from the same population? | Yes  No  Unclear  Not applicable | 1. and 2. No exposure group comparison  4. and 5. Confounding factors were not considered as the study is descriptive and does not compare exposure groups.  7. While 29 (1.17%) patients had their tissue retransplantated on site, 95 patients (3.83%) had their tissue outsources for scheduled retransplantation at 12 different referral centres.  8. follow-up time was depicted as storage duration (years) and grouped by storage time in: ≥5 years active storage n=661 (median storage duration 7.4 years) (range 5-16.3 years), ≥10 years active storage n=148 (median storage duration 11.9 years (range 10-16.3 years), ended storage n=1155 (median storage duration 3.8 years (range 0-19.1 years). No information of minimum-follow up time.  10. No sensitivity analysis was reported for incomplete follow-up/deceased patients. |
|  | 2. Were the exposures measured similarly to assign people to both exposed and unexposed groups? | Yes  No  Unclear  Not applicable |  |
|  | 3. Was the exposure measured in a valid and reliable way? | Yes  No  Unclear  Not applicable |  |
|  | 4. Were confounding factors identified? | Yes  No  Unclear  Not applicable |  |
|  | 5. Were strategies to deal with confounding factors stated? | Yes  No  Unclear  Not applicable |  |
|  | 6. Were the groups/participants free of the outcome at the start of the study (or at the moment of exposure)? | Yes  No  Unclear  Not applicable |  |
|  | 7. Were the outcomes measured in a valid and reliable way? | Yes  No  Unclear  Not applicable |  |
|  | 8. Was the follow up time reported and sufficient to be long enough for outcomes to occur? | Yes  No  Unclear  Not applicable |  |
|  | 9. Was follow up complete, and if not, were the reasons for loss to follow up described and explored? | Yes  No  Unclear  Not applicable |  |
|  | 10. Were strategies to address incomplete follow-up utilized? | Yes  No  Unclear  Not applicable |  |
|  | 11. Was appropriate statistical analysis used? | Yes  No  Unclear  Not applicable |  |
| Tanbo et al.  2015  Norway | 1. Were the two groups similar and recruited from the same population? | Yes  No  Unclear  Not applicable | 1.-2. No exposure group comparison  4.-5. Confounding factors were not considered as the study is descriptive and does not compare exposure groups.  8-9. Follow-up time not specified.  10. No sensitivity analysis was reported for incomplete follow-up/deceased patients. |
|  | 2. Were the exposures measured similarly to assign people to both exposed and unexposed groups? | Yes  No  Unclear  Not applicable |  |
|  | 3. Was the exposure measured in a valid and reliable way? | Yes  No  Unclear  Not applicable |  |
|  | 4. Were confounding factors identified? | Yes  No  Unclear  Not applicable |  |
|  | 5. Were strategies to deal with confounding factors stated? | Yes  No  Unclear  Not applicable |  |
|  | 6. Were the groups/participants free of the outcome at the start of the study (or at the moment of exposure)? | Yes  No  Unclear  Not applicable |  |
|  | 7. Were the outcomes measured in a valid and reliable way? | Yes  No  Unclear  Not applicable |  |
|  | 8. Was the follow up time reported and sufficient to be long enough for outcomes to occur? | Yes  No  Unclear  Not applicable |  |
|  | 9. Was follow up complete, and if not, were the reasons for loss to follow up described and explored? | Yes  No  Unclear  Not applicable |  |
|  | 10. Were strategies to address incomplete follow-up utilized? | Yes  No  Unclear  Not applicable |  |
|  | 11. Was appropriate statistical analysis used? | Yes  No  Unclear  Not applicable |  |
| Hoekman et al.  2020  Netherlands | 1. Were the two groups similar and recruited from the same population? | Yes  No  Unclear  Not applicable | 1.-2. No exposure group comparison  4.-5. Confounding factors were not considered as the study is descriptive and does not compare exposure groups.  8. Mean follow-up time 77.4 months (range 4-183 months). Return for OTT may occur several years after OTC and insufficient time may therefore bias the results.  10. No sensitivity analysis was reported for incomplete follow-up/deceased patients. |
|  | 2. Were the exposures measured similarly to assign people to both exposed and unexposed groups? | Yes  No  Unclear  Not applicable |  |
|  | 3. Was the exposure measured in a valid and reliable way? | Yes  No  Unclear  Not applicable |  |
|  | 4. Were confounding factors identified? | Yes  No  Unclear  Not applicable |  |
|  | 5. Were strategies to deal with confounding factors stated? | Yes  No  Unclear  Not applicable |  |
|  | 6. Were the groups/participants free of the outcome at the start of the study (or at the moment of exposure)? | Yes  No  Unclear  Not applicable |  |
|  | 7. Were the outcomes measured in a valid and reliable way? | Yes  No  Unclear  Not applicable |  |
|  | 8. Was the follow up time reported and sufficient to be long enough for outcomes to occur? | Yes  No  Unclear  Not applicable |  |
|  | 9. Was follow up complete, and if not, were the reasons for loss to follow up described and explored? | Yes  No  Unclear  Not applicable |  |
|  | 10. Were strategies to address incomplete follow-up utilized? | Yes  No  Unclear  Not applicable |  |
|  | 11. Was appropriate statistical analysis used? | Yes  No  Unclear  Not applicable |  |
| Imbert et al.  2014  Belgium | 1. Were the two groups similar and recruited from the same population? | Yes  No  Unclear  Not applicable | 1.-2. No exposure group comparison  4.-5. Confounding factors were not considered as the study is descriptive and does not compare exposure groups.  8-9. Follow-up time not specified.  10. No sensitivity analysis was reported for incomplete follow-up/deceased patients. |
|  | 2. Were the exposures measured similarly to assign people to both exposed and unexposed groups? | Yes  No  Unclear  Not applicable |  |
|  | 3. Was the exposure measured in a valid and reliable way? | Yes  No  Unclear  Not applicable |  |
|  | 4. Were confounding factors identified? | Yes  No  Unclear  Not applicable |  |
|  | 5. Were strategies to deal with confounding factors stated? | Yes  No  Unclear  Not applicable |  |
|  | 6. Were the groups/participants free of the outcome at the start of the study (or at the moment of exposure)? | Yes  No  Unclear  Not applicable |  |
|  | 7. Were the outcomes measured in a valid and reliable way? | Yes  No  Unclear  Not applicable |  |
|  | 8. Was the follow up time reported and sufficient to be long enough for outcomes to occur? | Yes  No  Unclear  Not applicable |  |
|  | 9. Was follow up complete, and if not, were the reasons for loss to follow up described and explored? | Yes  No  Unclear  Not applicable |  |
|  | 10. Were strategies to address incomplete follow-up utilized? | Yes  No  Unclear  Not applicable |  |
|  | 11. Was appropriate statistical analysis used? | Yes  No  Unclear  Not applicable |  |
| Hulsbosch et al.  2018  Belgium | 1. Were the two groups similar and recruited from the same population? | Yes  No  Unclear  Not applicable | 1. And 2. Although the original study included both OTC and GnRH agonist, only data regarding OTC were extracted for this review. No comparison of exposures for the purpose of this review was performed.  4. and 5. Confounding factors were not considered as the study is descriptive and does not compare exposure groups.  8. Follow-up was ≥3 years after the end of primary anticancer treatment. Mean follow-up time 61.5 months. Return for OTT may occur several years after OTC and insufficient time may therefore bias the results.  10. No sensitivity analysis was reported for incomplete follow-up/deceased patients. |
|  | 2. Were the exposures measured similarly to assign people to both exposed and unexposed groups? | Yes  No  Unclear  Not applicable |  |
|  | 3. Was the exposure measured in a valid and reliable way? | Yes  No  Unclear  Not applicable |  |
|  | 4. Were confounding factors identified? | Yes  No  Unclear  Not applicable |  |
|  | 5. Were strategies to deal with confounding factors stated? | Yes  No  Unclear  Not applicable |  |
|  | 6. Were the groups/participants free of the outcome at the start of the study (or at the moment of exposure)? | Yes  No  Unclear  Not applicable |  |
|  | 7. Were the outcomes measured in a valid and reliable way? | Yes  No  Unclear  Not applicable |  |
|  | 8. Was the follow up time reported and sufficient to be long enough for outcomes to occur? | Yes  No  Unclear  Not applicable |  |
|  | 9. Was follow up complete, and if not, were the reasons for loss to follow up described and explored? | Yes  No  Unclear  Not applicable |  |
|  | 10. Were strategies to address incomplete follow-up utilized? | Yes  No  Unclear  Not applicable |  |
|  | 11. Was appropriate statistical analysis used? | Yes  No  Unclear  Not applicable |  |
| Fabbri et al.  2022  Italy | 1. Were the two groups similar and recruited from the same population? | Yes  No  Unclear  Not applicable | 1.-2. No exposure group comparison  4.-5. Confounding factors were not considered as the study is descriptive and does not compare exposure groups.  8. Follow-up time not specified. However, the study describes 20 years’ experience pf OTC. Return for OTT may occur several years after OTC and insufficient time may therefore bias the results.  10. No sensitivity analysis was reported for incomplete follow-up/deceased patients. |
|  | 2. Were the exposures measured similarly to assign people to both exposed and unexposed groups? | Yes  No  Unclear  Not applicable |  |
|  | 3. Was the exposure measured in a valid and reliable way? | Yes  No  Unclear  Not applicable |  |
|  | 4. Were confounding factors identified? | Yes  No  Unclear  Not applicable |  |
|  | 5. Were strategies to deal with confounding factors stated? | Yes  No  Unclear  Not applicable |  |
|  | 6. Were the groups/participants free of the outcome at the start of the study (or at the moment of exposure)? | Yes  No  Unclear  Not applicable |  |
|  | 7. Were the outcomes measured in a valid and reliable way? | Yes  No  Unclear  Not applicable |  |
|  | 8. Was the follow up time reported and sufficient to be long enough for outcomes to occur? | Yes  No  Unclear  Not applicable |  |
|  | 9. Was follow up complete, and if not, were the reasons for loss to follow up described and explored? | Yes  No  Unclear  Not applicable |  |
|  | 10. Were strategies to address incomplete follow-up utilized? | Yes  No  Unclear  Not applicable |  |
|  | 11. Was appropriate statistical analysis used? | Yes  No  Unclear  Not applicable |  |
| Silber et al.  2022  USA | 1. Were the two groups similar and recruited from the same population? | Yes  No  Unclear  Not applicable | 1.-2. No exposure group comparison  4.-5. Confounding factors were not considered as the study is descriptive and does not compare exposure groups.  8-9. Follow-up time not specified.  10. No sensitivity analysis was reported for incomplete follow-up/deceased patients. |
|  | 2. Were the exposures measured similarly to assign people to both exposed and unexposed groups? | Yes  No  Unclear  Not applicable |  |
|  | 3. Was the exposure measured in a valid and reliable way? | Yes  No  Unclear  Not applicable |  |
|  | 4. Were confounding factors identified? | Yes  No  Unclear  Not applicable |  |
|  | 5. Were strategies to deal with confounding factors stated? | Yes  No  Unclear  Not applicable |  |
|  | 6. Were the groups/participants free of the outcome at the start of the study (or at the moment of exposure)? | Yes  No  Unclear  Not applicable |  |
|  | 7. Were the outcomes measured in a valid and reliable way? | Yes  No  Unclear  Not applicable |  |
|  | 8. Was the follow up time reported and sufficient to be long enough for outcomes to occur? | Yes  No  Unclear  Not applicable |  |
|  | 9. Was follow up complete, and if not, were the reasons for loss to follow up described and explored? | Yes  No  Unclear  Not applicable |  |
|  | 10. Were strategies to address incomplete follow-up utilized? | Yes  No  Unclear  Not applicable |  |
|  | 11. Was appropriate statistical analysis used? | Yes  No  Unclear  Not applicable |  |
| Choi et al.  2022  Korea | 1. Were the two groups similar and recruited from the same population? | Yes  No  Unclear  Not applicable | 1. And 2. Although the original study included both OTC, OC and EC, only data regarding OTC were extracted for this review. No comparison of exposures for the purpose of this review was performed.  4. and 5. Confounding factors were not considered as the study is descriptive and does not compare exposure groups.  8. mean duration of the cryopreserved ovarian tissue was 4.98 (±2.85) years and the longest was stored for 12.5 years. No information of minimum FU-time. Return for OTT may occur several years after OTC and insufficient time may therefore bias the results.  9. No information of loss-to-follow-up, also no information of number of deceased patients.  10. No sensitivity analysis was reported for incomplete follow-up/deceased patients. |
|  | 2. Were the exposures measured similarly to assign people to both exposed and unexposed groups? | Yes  No  Unclear  Not applicable |  |
|  | 3. Was the exposure measured in a valid and reliable way? | Yes  No  Unclear  Not applicable |  |
|  | 4. Were confounding factors identified? | Yes  No  Unclear  Not applicable |  |
|  | 5. Were strategies to deal with confounding factors stated? | Yes  No  Unclear  Not applicable |  |
|  | 6. Were the groups/participants free of the outcome at the start of the study (or at the moment of exposure)? | Yes  No  Unclear  Not applicable |  |
|  | 7. Were the outcomes measured in a valid and reliable way? | Yes  No  Unclear  Not applicable |  |
|  | 8. Was the follow up time reported and sufficient to be long enough for outcomes to occur? | Yes  No  Unclear  Not applicable |  |
|  | 9. Was follow up complete, and if not, were the reasons for loss to follow up described and explored? | Yes  No  Unclear  Not applicable |  |
|  | 10. Were strategies to address incomplete follow-up utilized? | Yes  No  Unclear  Not applicable |  |
|  | 11. Was appropriate statistical analysis used? | Yes  No  Unclear  Not applicable |  |
| Sánchez et al.  2008  Valencia | 1. Were the two groups similar and recruited from the same population? | Yes  No  Unclear  Not applicable | 1.-2. No exposure group comparison  4.-5. Confounding factors were not considered as the study is descriptive and does not compare exposure groups.  8. No information of follow.up time. Also no information of time period for OTC.  10. No sensitivity analysis was reported for incomplete follow-up/deceased patients. |
|  | 2. Were the exposures measured similarly to assign people to both exposed and unexposed groups? | Yes  No  Unclear  Not applicable |  |
|  | 3. Was the exposure measured in a valid and reliable way? | Yes  No  Unclear  Not applicable |  |
|  | 4. Were confounding factors identified? | Yes  No  Unclear  Not applicable |  |
|  | 5. Were strategies to deal with confounding factors stated? | Yes  No  Unclear  Not applicable |  |
|  | 6. Were the groups/participants free of the outcome at the start of the study (or at the moment of exposure)? | Yes  No  Unclear  Not applicable |  |
|  | 7. Were the outcomes measured in a valid and reliable way? | Yes  No  Unclear  Not applicable |  |
|  | 8. Was the follow up time reported and sufficient to be long enough for outcomes to occur? | Yes  No  Unclear  Not applicable |  |
|  | 9. Was follow up complete, and if not, were the reasons for loss to follow up described and explored? | Yes  No  Unclear  Not applicable |  |
|  | 10. Were strategies to address incomplete follow-up utilized? | Yes  No  Unclear  Not applicable |  |
|  | 11. Was appropriate statistical analysis used? | Yes  No  Unclear  Not applicable |  |
| Oktay and Oktem  2010  USA | 1. Were the two groups similar and recruited from the same population? | Yes  No  Unclear  Not applicable | 1.-2. No exposure group comparison  4.-5. Confounding factors were not considered as the study is descriptive and does not compare exposure groups.  8. Median length of storage was 3.06 (±0.2) years (0.05-10.5 years. Furthermore, return for OTT may occur several years after OTC and insufficient time may therefore bias the results.  10. No sensitivity analysis was reported for incomplete follow-up/deceased patients. |
|  | 2. Were the exposures measured similarly to assign people to both exposed and unexposed groups? | Yes  No  Unclear  Not applicable |  |
|  | 3. Was the exposure measured in a valid and reliable way? | Yes  No  Unclear  Not applicable |  |
|  | 4. Were confounding factors identified? | Yes  No  Unclear  Not applicable |  |
|  | 5. Were strategies to deal with confounding factors stated? | Yes  No  Unclear  Not applicable |  |
|  | 6. Were the groups/participants free of the outcome at the start of the study (or at the moment of exposure)? | Yes  No  Unclear  Not applicable |  |
|  | 7. Were the outcomes measured in a valid and reliable way? | Yes  No  Unclear  Not applicable |  |
|  | 8. Was the follow up time reported and sufficient to be long enough for outcomes to occur? | Yes  No  Unclear  Not applicable |  |
|  | 9. Was follow up complete, and if not, were the reasons for loss to follow up described and explored? | Yes  No  Unclear  Not applicable |  |
|  | 10. Were strategies to address incomplete follow-up utilized? | Yes  No  Unclear  Not applicable |  |
|  | 11. Was appropriate statistical analysis used? | Yes  No  Unclear  Not applicable |  |
| Delattre et al.  2020  Belgium | 1. Were the two groups similar and recruited from the same population? | Yes  No  Unclear  Not applicable | 1. And 2. Although the original study included various FP procedures, either stand alone or combined, only data regarding OTC were extracted for this review. No comparison of exposures for the purpose of this review was performed.  4. and 5. Confounding factors were not considered as the study is descriptive and does not compare exposure groups.  7. As the women received a combination of FP, and may have opted for returning to use the other FP treatments before OTT, the return rate may be biased.  8. Follow up described as “during the timeframe of the study” which was from January 2012-december 2018. Return for OTT may occur several years after OTC and insufficient time may therefore bias the results.  10. No sensitivity analysis was reported for incomplete follow-up/deceased patients. |
|  | 2. Were the exposures measured similarly to assign people to both exposed and unexposed groups? | Yes  No  Unclear  Not applicable |  |
|  | 3. Was the exposure measured in a valid and reliable way? | Yes  No  Unclear  Not applicable |  |
|  | 4. Were confounding factors identified? | Yes  No  Unclear  Not applicable |  |
|  | 5. Were strategies to deal with confounding factors stated? | Yes  No  Unclear  Not applicable |  |
|  | 6. Were the groups/participants free of the outcome at the start of the study (or at the moment of exposure)? | Yes  No  Unclear  Not applicable |  |
|  | 7. Were the outcomes measured in a valid and reliable way? | Yes  No  Unclear  Not applicable |  |
|  | 8. Was the follow up time reported and sufficient to be long enough for outcomes to occur? | Yes  No  Unclear  Not applicable |  |
|  | 9. Was follow up complete, and if not, were the reasons for loss to follow up described and explored? | Yes  No  Unclear  Not applicable |  |
|  | 10. Were strategies to address incomplete follow-up utilized? | Yes  No  Unclear  Not applicable |  |
|  | 11. Was appropriate statistical analysis used? | Yes  No  Unclear  Not applicable |  |
| Yap and Davies  2007  UK | 1. Were the two groups similar and recruited from the same population? | Yes  No  Unclear  Not applicable | 1. And 2. Although the original study included both OTC, OC and EC as FP, only data regarding OTC were extracted for this review. No comparison of exposures for the purpose of this review was performed.  4. and 5. Confounding factors were not considered as the study is descriptive and does not compare exposure groups.  8. Follow-up time was not specified.  10. No sensitivity analysis was reported for incomplete follow-up/deceased patients. |
|  | 2. Were the exposures measured similarly to assign people to both exposed and unexposed groups? | Yes  No  Unclear  Not applicable |  |
|  | 3. Was the exposure measured in a valid and reliable way? | Yes  No  Unclear  Not applicable |  |
|  | 4. Were confounding factors identified? | Yes  No  Unclear  Not applicable |  |
|  | 5. Were strategies to deal with confounding factors stated? | Yes  No  Unclear  Not applicable |  |
|  | 6. Were the groups/participants free of the outcome at the start of the study (or at the moment of exposure)? | Yes  No  Unclear  Not applicable |  |
|  | 7. Were the outcomes measured in a valid and reliable way? | Yes  No  Unclear  Not applicable |  |
|  | 8. Was the follow up time reported and sufficient to be long enough for outcomes to occur? | Yes  No  Unclear  Not applicable |  |
|  | 9. Was follow up complete, and if not, were the reasons for loss to follow up described and explored? | Yes  No  Unclear  Not applicable |  |
|  | 10. Were strategies to address incomplete follow-up utilized? | Yes  No  Unclear  Not applicable |  |
|  | 11. Was appropriate statistical analysis used? | Yes  No  Unclear  Not applicable |  |
| Takae et al.  2023  Japan | 1. Were the two groups similar and recruited from the same population? | Yes  No  Unclear  Not applicable | 1. And 2. Although the original study included both various FP methods, only data regarding OTC were extracted for this review. No comparison of exposures for the purpose of this review was performed.  4. and 5. Confounding factors were not considered as the study is descriptive and does not compare exposure groups.  7. Mailed-in questionnaire survey regarding number of OTC and OTT, amongst others, was sent to facilities offering OTC as FP. 43:51 responded, and 18:43 facilities had performed OTC.  8. FU time not specified.  10. No sensitivity analysis was reported for incomplete follow-up/deceased patients. |
|  | 2. Were the exposures measured similarly to assign people to both exposed and unexposed groups? | Yes  No  Unclear  Not applicable |  |
|  | 3. Was the exposure measured in a valid and reliable way? | Yes  No  Unclear  Not applicable |  |
|  | 4. Were confounding factors identified? | Yes  No  Unclear  Not applicable |  |
|  | 5. Were strategies to deal with confounding factors stated? | Yes  No  Unclear  Not applicable |  |
|  | 6. Were the groups/participants free of the outcome at the start of the study (or at the moment of exposure)? | Yes  No  Unclear  Not applicable |  |
|  | 7. Were the outcomes measured in a valid and reliable way? | Yes  No  Unclear  Not applicable |  |
|  | 8. Was the follow up time reported and sufficient to be long enough for outcomes to occur? | Yes  No  Unclear  Not applicable |  |
|  | 9. Was follow up complete, and if not, were the reasons for loss to follow up described and explored? | Yes  No  Unclear  Not applicable |  |
|  | 10. Were strategies to address incomplete follow-up utilized? | Yes  No  Unclear  Not applicable |  |
|  | 11. Was appropriate statistical analysis used? | Yes  No  Unclear  Not applicable |  |
| Takae et al.  2022  Japan | 1. Were the two groups similar and recruited from the same population? | Yes  No  Unclear  Not applicable | 1. And 2. Although the original study included both various FP methods, only data regarding OTC were extracted for this review. No comparison of exposures for the purpose of this review was performed.  4. and 5. Confounding factors were not considered as the study is descriptive and does not compare exposure groups.  7. Mailed-in questionnaire survey regarding number of OTC and OTT, amongst others, was sent to facilities offering OTC as FP. 30:30 responded.  8. Follow-up time not specified.  10. No sensitivity analysis was reported for incomplete follow-up/deceased patients. |
|  | 2. Were the exposures measured similarly to assign people to both exposed and unexposed groups? | Yes  No  Unclear  Not applicable |  |
|  | 3. Was the exposure measured in a valid and reliable way? | Yes  No  Unclear  Not applicable |  |
|  | 4. Were confounding factors identified? | Yes  No  Unclear  Not applicable |  |
|  | 5. Were strategies to deal with confounding factors stated? | Yes  No  Unclear  Not applicable |  |
|  | 6. Were the groups/participants free of the outcome at the start of the study (or at the moment of exposure)? | Yes  No  Unclear  Not applicable |  |
|  | 7. Were the outcomes measured in a valid and reliable way? | Yes  No  Unclear  Not applicable |  |
|  | 8. Was the follow up time reported and sufficient to be long enough for outcomes to occur? | Yes  No  Unclear  Not applicable |  |
|  | 9. Was follow up complete, and if not, were the reasons for loss to follow up described and explored? | Yes  No  Unclear  Not applicable |  |
|  | 10. Were strategies to address incomplete follow-up utilized? | Yes  No  Unclear  Not applicable |  |
|  | 11. Was appropriate statistical analysis used? | Yes  No  Unclear  Not applicable |  |
| Finkelstein et al.  2025  Australia | 1. Were the two groups similar and recruited from the same population? | Yes  No  Unclear  Not applicable | 1.-2. No exposure group comparison  4.-5. Confounding factors were not considered as the study is descriptive and does not compare exposure groups.  8. Follow-up time not specified.  10. No sensitivity analysis was reported for incomplete follow-up/deceased patients. |
|  | 2. Were the exposures measured similarly to assign people to both exposed and unexposed groups? | Yes  No  Unclear  Not applicable |  |
|  | 3. Was the exposure measured in a valid and reliable way? | Yes  No  Unclear  Not applicable |  |
|  | 4. Were confounding factors identified? | Yes  No  Unclear  Not applicable |  |
|  | 5. Were strategies to deal with confounding factors stated? | Yes  No  Unclear  Not applicable |  |
|  | 6. Were the groups/participants free of the outcome at the start of the study (or at the moment of exposure)? | Yes  No  Unclear  Not applicable |  |
|  | 7. Were the outcomes measured in a valid and reliable way? | Yes  No  Unclear  Not applicable |  |
|  | 8. Was the follow up time reported and sufficient to be long enough for outcomes to occur? | Yes  No  Unclear  Not applicable |  |
|  | 9. Was follow up complete, and if not, were the reasons for loss to follow up described and explored? | Yes  No  Unclear  Not applicable |  |
|  | 10. Were strategies to address incomplete follow-up utilized? | Yes  No  Unclear  Not applicable |  |
|  | 11. Was appropriate statistical analysis used? | Yes  No  Unclear  Not applicable |  |
| Beckmann et al.  2018  FertiProtekt (Germany, Austria, Switzerland) | 1. Were the two groups similar and recruited from the same population? | Yes  No  Unclear  Not applicable | 1. and 2. No exposure group comparison  4. and 5. Confounding factors were not considered as the study is descriptive and does not compare exposure groups.  7. This was a multicenter study including only centers within the FertiProtect network that had performed at least five OTC and three OTT procedures. It is not specified whether any centers were excluded for not meeting these criteria.  8., 9. and 10. OTT was a secondary outcome. No information of FU time was available. |
|  | 2. Were the exposures measured similarly to assign people to both exposed and unexposed groups? | Yes  No  Unclear  Not applicable |  |
|  | 3. Was the exposure measured in a valid and reliable way? | Yes  No  Unclear  Not applicable |  |
|  | 4. Were confounding factors identified? | Yes  No  Unclear  Not applicable |  |
|  | 5. Were strategies to deal with confounding factors stated? | Yes  No  Unclear  Not applicable |  |
|  | 6. Were the groups/participants free of the outcome at the start of the study (or at the moment of exposure)? | Yes  No  Unclear  Not applicable |  |
|  | 7. Were the outcomes measured in a valid and reliable way? | Yes  No  Unclear  Not applicable |  |
|  | 8. Was the follow up time reported and sufficient to be long enough for outcomes to occur? | Yes  No  Unclear  Not applicable |  |
|  | 9. Was follow up complete, and if not, were the reasons for loss to follow up described and explored? | Yes  No  Unclear  Not applicable |  |
|  | 10. Were strategies to address incomplete follow-up utilized? | Yes  No  Unclear  Not applicable |  |
|  | 11. Was appropriate statistical analysis used? | Yes  No  Unclear  Not applicable |  |
